# Opening the black box: insights into ubiquitin-mediated control of innate antiviral immunity and AI-enhanced therapeutics

**DOI:** 10.3389/fimmu.2026.1869543

**Published:** 2026-06-15

**Authors:** Manasvi Jagannath, Ananya Sunderesan, Manshi Kumari Gupta, C. Sudandiradoss

**Affiliations:** Department of Biotechnology, School of Bio Sciences and Technology, Vellore Institute of Technology, Vellore, Tamil Nadu, India

**Keywords:** AI/ML, antiviral immunity, black box, therapeutics, ubiquitination

## Abstract

Innate antiviral immunity serves as the first line of defence against viral infections by detecting viruses and triggering responses to eliminate infected cells. However, viruses often evade such host defence mechanisms by hijacking essential post-translational modifications (PTMs) involved in these pathways, particularly ubiquitination, which plays a central role in immune signalling and targeted degradation. This makes ubiquitin sites and E3 ligases key targets for developing degradative antiviral therapeutics. Both have been studied extensively for degradative drugs such as Proteolysis Targeting Chimeras (PROTACs), but their antiviral potential still requires further investigation. Parallel advancements in artificial intelligence have transformed drug design, enabling precise *de novo* design and PTM site predictions, yet AI-based, ubiquitin-centred antiviral strategies remain underexplored. In this review, we connect these areas by examining the role of ubiquitination in antiviral immunity and viral evasion, identifying limitations in current AI-driven approaches, and outlining how AI can be leveraged to design targeted antiviral therapies. We further discuss the need for explainable AI to address interpretability challenges and consider the risks of AI use in healthcare. Finally, we highlight the potential of integrating structural and emerging 4D modelling approaches to better understand dynamic viral protein complexes and support next-generation antiviral design.

## 1 Introduction

Innate immune responses are triggered when a foreign antigen is detected by pattern recognition receptors, which recruit immune cells to purge the pathogen from the body. For viruses specifically, receptors recognise fragments of viral DNA or RNA to recruit immune cells and transcription factors for antiviral genes to trigger interferon signalling (1). This interferon cascade leads to autophagy of infected cells and detected viral proteins, facilitated by ubiquitin tags, which mark them for proteasomal degradation (2). Ubiquitination plays a key role in regulating these antiviral responses because ubiquitin tags act as markers with twofold functions: (a) to link proteins for recruitment of interferon regulatory factors and (b) to mark pattern recognition receptors for degradation to prevent a prolonged inflammatory immune response (3). Viruses manipulate this sequence of events in various ways to evade host antiviral responses. Two such examples are molecular mimicry and the use of viral deubiquitinases (DUBs).

Viral evasion tactics have been a hurdle in designing potent and target-specific antiviral drugs, primarily because viral motifs tend to closely resemble host proteins, posing the threat of targeting and destroying host cells or essential proteins (4). However, over recent years, structural modelling has advanced significantly with the use of artificial intelligence, leading to better predictions, more robust drug-targeting, and more effective structure-guided drug design (5). The classic problems with antiviral drug design are the structural uncertainty of viral proteins and complexes, the time-consuming process of experimental analyses like cryo-EM, and the quick mutation of viruses that leads to drug resistance (6). AI-based structure prediction models, such as AlphaFold, address some of these problems quite well, with rapid and highly accurate predictions of viral-host protein interactions and protein-protein interfaces (7).

Generative models (such as Generative Adversarial Networks or GANs) enable the design of target-specific viral inhibitors (such as antiviral peptides) by exploring their chemical space via reinforcement learning (RL), which allows potential candidates to be optimized for specific antiviral properties (8, 9). AI is also being used to optimize targeted protein degradation via the host’s ubiquitin-proteasome system (UPS) with advances in the design of PROTACs (Proteolysis-Targeting Chimeras) and MGDs (Molecular Glue Degraders) (10). Both induce conformational changes in UPS-associated enzymes that are hijacked by viruses (mainly host E3 ligases) to bring them closer to viral targets, enabling proteolytic degradation. PROTACs attach to E3 ligase ligands via a linker molecule, bringing the ligase closer to the viral protein target (11), while MGDs do so by disrupting the viral-host interface without an additional linker.

While AI-based therapeutic design is a highly attractive means of drug discovery, there are limitations associated with AI-generated models and predictions, which make it difficult to utilise these predictions practically. For instance, the classic “Black Box” problem arises due to the lack of transparency in AI models, where it is unclear how predictions are made. This makes them hard to interpret and validate biologically. Deep Neural Networks are designed to perform certain tasks beyond human ability, such as quickly analysing large datasets to find meaningful trends and patterns (12). However, this often means the predictions cannot be validated because the reasoning behind them is beyond human comprehension (13). Recent advancements in explainable AI (XAI) have made it a potential solution to this problem (14). Generative AI models also pose the risk of “hallucinating” and producing incorrect outputs (15). It is being theorised that this can be solved by forcing the AI to run its predictions through verified data before providing an output. A significant cause for unreliable predictions from AI models is the lack of available therapeutic data and the small size of existing datasets. Existing training datasets are not large enough for robust predictions and often lead to significant overfitting (16). They also lack diversity and uniformity, which leads to biased models that perform poorly with underrepresented viral clades or host genotypes, with no way of evaluating this bias (17, 18).

Over the years, there has been extensive research and review surrounding ubiquitin-mediated antiviral signalling, viral evasion mechanisms, and AI-based therapeutics. However, these three fields have been explored in isolation, with little insight into their points of intersection. Given ubiquitin’s essential regulatory role in antiviral signalling pathways and the immense potential of AI-based antiviral therapeutics, we aim to integrate all three areas of study. Our three main objectives are (i) to highlight the importance of ubiquitination in antiviral immunity and viral evasion, (ii) to analyse the role of AI in ubiquitin-driven therapeutics and drug design (iii) to identify key challenges in applying AI to ubiquitin-targeted antivirals and propose future directions to overcome them.

## 2 The molecular arms race: mechanisms of immunity and evasion

### 2.1 Regulation of viral RNA and DNA sensing

All infecting pathogens leave behind traces called pathogen-associated molecular patterns (PAMPs), which are identified by host pattern recognition receptors (PRRs), triggering innate immune responses. Viral PAMPs can often contain structurally conserved RNA or DNA fragments, which are sensed by three pathways in host cells, based on the PRRs involved (19). The three most common types of PRRs involved in innate antiviral immunity are Toll-like receptors (TLRs), retinoic acid-inducible gene I (RIG-I)-like receptors (RLRs), and cyclic GMP-AMP synthase (cGAS) stimulator of interferon genes (STING). All three converge at one point: a signalling cascade producing proinflammatory cytokines and type 1 interferons (IFN-I) to clear out the virus (20).

TLRs have particularly high expression levels in immune cells and are involved in both DNA and RNA sensing. They can be classified based on their location, with TLRs 1/2 and 2/6 existing as cell surface heterodimers, while TLRs 3, 7, 8, and 9 are expressed in endosomes. They require Toll/interleukin-1 receptor (TIR) domain-containing adaptors that have two functions, bridging and signalling (21). TIR domain-containing adaptor protein inducing interferon beta (TRIF) is one such signalling adaptor which works with TRIF-related adaptor molecule (TRAM) in the initial stages of TLR signalling (22). All TLRs, except for TLR3, utilize this combination of adaptors, with TLR3 using TRIF alone to activate transcription factors, interferon regulatory factor 3 (IRF3) and NF-κB, leading to viral antigen-specific T cell responses (23).

RLRs are sensors of viral RNA present in the host’s cytoplasm. They are found on proteins of the RNA helicase family (common examples are RIG-I and MDA5 (melanoma differentiation-associated protein 5)) (24). These RLR helicases contain four essential domains: two helicase domains (Hel1 and Hel2) and two N-terminal caspase activation and recruitment domains (CARDs). The helicase domains have ATP-dependant interactions with RNA ligands, making them key for RNA sensing, while the CARDs mediate downstream signalling by associating with the adaptor, mitochondrial antiviral-signalling protein (MAVS) (25). MAVS also contains an N-terminal CARD domain to facilitate this forming signal-competent aggregates involved in the recruitment of downstream signalling molecules (TNF receptor- associated factor (TRAF)3/6 and IKK family members like IKKϵ, TBK1) leading to enhanced production of IFN (26).

cGAS is considered a cytosolic PRR due to its ability to sense cytosolic viral dsDNA, however its role begins in the nucleus, where its tightly tethered to chromatin and remains inactive until viral infection (27). Infecting viruses cause replicative stress that alters chromosome structure, and breaks the nuclear envelope, releasing cGAS from its tether. Since most DNA viruses replicate within the nucleus during infection, cGAS is primed for their detection (28). Once it detects dsDNA, it catalyses the formation of the STING signalosome (cGAMP bound to STING) which recruits TBK1 and is then trafficked by the endoplasmic reticulum. This activates both IRF3 and NF-κB, triggering the production of IFN-I and proinflammatory cytokines (29).

All three signalling pathways drive IFN-I production to clear viral infection, starting at different points and converging on the same end. TRIF (endosomal TLR3 and TLR4), MAVS (RIG-I) and STING (cGAS) are all adaptors phosphorylated by inhibitor of NF-κB kinase (IKK) related kinases (20). TANK-binding kinase 1 (TBK1) and IKKϵ are two common examples. This phosphorylation recruits interferon regulatory factors (such as IRF3 and IRF7), which then dimerize, driving NF-κB activation and consequential IFN production (30).

TLR, RLR, and cGAS signalling pathways all have individual dominance over a specific type of nucleic acid sensing, with RLRs detecting cytosolic RNA, TLRs detecting endosomal PAMPs, and cGAS sensing cytosolic DNA. However, some amount of crosstalk between all three does take place to enhance interferon (IFN) signalling (31).

Since these sensing pathways depend on rapid signal propagation and timely termination, their activity is tightly regulated by ubiquitin-mediated modifications.

### 2.2 Ubiquitin-mediated PRR signalling activation

PRR Signalling (pattern recognition receptor signalling) refers to a series of pathways in the immune system that are mainly involved in immune defence and regulation. These pathways often require fine-tuning via post-translational modifications and recruitment of inhibitors or amplifiers for precise targeting (32). Ubiquitination is one such post-translational modification that plays a key role in innate antiviral immune responses through two main processes: K63 chain-linked scaffolding and K48 chain-tagged proteasomal degradation (refer **Table 1** for other, atypical ubiquitin linkages).

**Table 1:** Ubiquitination and ubiquitin-like post translational modifications in antiviral PRR signalling.

PTM type	Functional role	Representative example	References
K63-linked Ubiquitination	Non-degradative signal involved in scaffold formation to activate IFN production	TRIM25 ubiquitinates RIG-1 to activate MAVS signalling.	(133)
K48-linked Ubiquitination	Degradative signal that regulates antiviral signalling cascades by tagging PRRs for proteasomal degradation	Triad3A ubiquitinates TRAF3 and TBK1 to moderate IFN-β production.	(134)
M1-linked (Linear) Ubiquitination	Regulatory signal which prevents overproduction of IFN	LUBAC attaches M1-linear chains to NEMO (NF-κB essential modulator) to recruit TBK1 and OTULIN hydrolyses them to disassemble the signalosome	(135)
K6-linked Ubiquitination	Non-degradative signal, enhancing antiviral gene transcription and amplify IFN production	TRIM65 boosts K6 ubiquitination of IRF3, increasing expression of IFN-β and ISGs.	(136)
K11-linked Ubiquitination	Regulation and proteasome-mediated degradation alongside K48-linked ubiquitination	USP19 removes K11 chains on Beclin-1, inducing autophagy and repressing RIG-1-MAVS interaction	(137)
K27-linked Ubiquitination	Non-degradative signal that activates transcription of IFNs and proinflammatory cytokines	TRIM21 ubiquitinates MAVS, activating NF-κB and leading to transcription of TNF-α and IL-6	(138)
ISGylation	Inhibits viral replication by conjugating to viral components and causing steric hindrance	ISGylation of the SARS-CoV-2 N protein to prevent its oligomerization reducing RNA synthesis	(139, 140)
SUMOylation	Stabilises and modulates ubiquitin-mediated processes such as NF-κB activation and proteasomal degradation of infected cells	PIAS1 conjugates SUMO1, 2, 3 to Influenza A viral PB2 polymerase, causing proteasomal degradation and restricting viral replication	(141, 142)
NEDDylation	Induces production of pro-inflammatory cytokines by initiating antiviral gene expression	Initiation of gene expression by NF-κB is dependent on neddylation of ubiquitin E3 ligase	(143)

This table classifies canonical and atypical ubiquitination and ubiquitin-associated PTMs by type (linked residues or molecules) and function (activating, degradative, regulatory) with examples across the RIG-I/MAVS, TLR and cGAS-STING viral sensing pathways. It showcases how canonical linkages (K63 and K48) drive signalosome assembly while atypical chains support and enhance these processes via complementary regulation of sensors and transcription factors.

K63 ubiquitin chains build a protein ‘scaffold’ by linking to adaptor proteins in viral sensing pathways (RIG-I, MAVS, STING, TRAF3), which subsequently results in the recruitment of kinases and interferon-regulatory factors to form a protein complex that activates an interferon signalling cascade (the immune defence response to viral detection) (33). For instance, STING activation after cGAS or cGAMP binding requires E3 ubiquitin ligases TRIM56 or TRIM32 to attach K63 chains to form docking sites for kinases (34). This also facilitates movement of the signalosome into the endoplasmic reticulum, where it recruits proteins for antiviral interferon signalling. Once in the ER, the STING signalosome recruits TBK1 (TANK-binding kinase 1), which begins type-1 interferon secretion (35).

Once K63 chains have induced signal activation, K48 ubiquitin chains are added to regulate and terminate the reaction. In antiviral immunity, this function of K48 chains facilitates expression of antiviral genes by inducing proteasomal degradation of the inhibitor for nuclear factor kappa B (or IκB) (36). The IκB kinase complex phosphorylates IκB, inducing K48-linked ubiquitination, which marks it for proteasomal degradation, allowing expression of NF κB. NF κB is an essential transcription factor for genes coding for antiviral cytokines (37). Similarly, K48 chains are attached to PRRs (such as RIG-I and TLR3) by negative-regulator E3 ligases (such as RNF125) to mark them for degradation (38). This terminates the antiviral pathway once the virus is cleared, avoiding interferon toxicity and septic shock.

In addition to ubiquitination, there are other ubiquitin-like post-translational modifications which have recently been discovered to interact with antiviral signals, such as the addition of SUMO (Small Ubiquitin-related Modifier) proteins or SUMOylation and the conjugation of ISGs (Interferon-Stimulated Genes) or ISGylation. While both these processes have been established as key parts of antiviral signalling, their precise mechanisms and outcomes are still vague and under research. SUMOylation sometimes inhibits the regulation of NF-κB by competing with ubiquitin to attach to the same lysine residues (39). On the contrary, ISG is associated with enhanced antiviral signalling, since it stabilizes active forms of antiviral signals (40). **Table 1** below summarizes the functional roles of canonical and atypical ubiquitination as well as ubiquitin-like PTMs in antiviral PRR signalling.

Although ubiquitination regulates a powerful immune response, the same process also makes certain host ubiquitinating enzymes targets for viral hijacking. TRIM29 is one such E3 ligase which moderates interferon signalling by promoting K48-linked degradation of STING and IKKγ (41) as well as K-11 linked degradation of MAVS (42). Viruses (such as Epstein Barr or rotavirus) upregulate the TRIM29 gene, limiting cytokine production and consequently promoting viral proliferation (43). Xing et al. (44) studied TRIM29 knockdown in human cell lines infected by the Epstein Barr Virus and Wang et al. (45) examined the role of TRIM29 in causing pathogenesis of viral myocarditis, with both studies producing results that support this claim. Xing et al. (46) showed that mouse models with TRIM29 deletions were protected against infection by the influenza virus. Together, these studies imply that TRIM29 and associated binding sites are promising targets for antiviral therapeutics, especially since AI frameworks now have the capability to both predict binding surfaces and design molecules to disrupt them, as discussed later on.

In summary, PRRs function by recognizing pathogen-associated molecular patterns (or PAMPs) and then triggering an immune response via a signalling cascade involving multiple interferon-regulatory factors, nuclear factors, and toll-like receptors. Within this process, K63 ubiquitin chains act as non-degradative links between proteins while K48 chains act as ‘tags’ for proteasomal degradation, terminating the PRR signal. Other atypical ubiquitin chains are also involved in innate immunity, such as K27, K6, and K11, however K63 and K48 are the most significant chains. This also makes them targets during viral evasion, when host E3 ligases are hijacked or erased.

### 2.3 Viral evasion by viral deubiquitinases

Viruses have evolved mechanisms to evade host immune responses by reversing the process of ubiquitin conjugation that is usually used by host cells for antiviral immunity. They do this by deploying enzymes known as viral deubiquitinases (vDUBs), which are viral proteases that cleave peptide or isopeptide bonds between ubiquitin and the substrate protein, thereby dismantling signalling scaffolds (47).

Viral DUBs have dual functions. Primarily, they act as viral proteases, enabling viral replication by cleaving viral polyproteins. However, due to evolution, they have acquired a secondary function, which is the ability to recognize and hydrolyse complex isopeptide bonds that link ubiquitin to host proteins. Viral DUBs act by removing ubiquitin residues from PRRs and their downstream adaptors. Specifically, these viral proteases disrupt the K63-linked and M1-linked ubiquitin scaffolds that are required to propagate signals through RLRs, TLRs, and the cGAS-STING pathways. Furthermore, vDUBs successfully inactivate the NF-κB signalling pathway by deubiquitinating central signalling hubs such as the TRAF complexes or NEMO. This deconjugation physically disrupts the structural platforms required for signal transduction, thereby blinding the host cell and silencing transcription of Type I interferons and pro-inflammatory cytokines, preventing full activation of host immune responses (48).

vDUBs are of several types, and their diversity can be clearly seen by considering two examples: the papain-like protease (PLpro) of SARS-CoV-2 and the ovarian tumour (OTU) domain of the Crimean-Congo Haemorrhagic Fever Virus (CCHFV).

PLpro shares structural homology with human Ubiquitin-Specific Proteases (USPs) and utilizes a classic right-handed “thumb-palm-fingers” architecture. It uses a precise loop to recognize a specific LXGG motif and heavily prefers cutting specific immune signals like ISG15 and K48 chains from STING, enabling the virus to bypass host defence (49, 50). In stark contrast to the USP-like fold of coronaviruses, the Crimean-Congo Haemorrhagic Fever Virus (CCHFV) utilizes an Ovarian Tumour (OTU) domain. Structural mapping reveals that the viral OTU has a unique N-terminal extension that forces the ubiquitin substrate to bind in an orientation rotated nearly 75 degrees. This results in the expansion of the catalytic cleft’s accessibility and grants the virus its promiscuous ability to rapidly hydrolyse both ubiquitin and ISG15, aiding in viral evasion (51).

However, erasing the ubiquitin code via vDUBs is only half of the evolutionary arms race. While vDUBs specialize in enzymatic subtraction, there is another non-catalytic strategy of viral evasion that involves hijacking E3 ligases to exemplify immune evasion through molecular mimicry and ligase redirection.

### 2.4 Viral evasion by molecular mimicry and ligase redirection

While viral deubiquitinases (vDUBs) disrupt immune signalling in a relatively passive manner by removing ubiquitin chains from host sensors, many viruses employ a more aggressive strategy known as molecular mimicry. In this mechanism, viruses encode proteins that structurally or functionally resemble components of the host ubiquitin machinery, such as E3 ligases, adaptor proteins, or substrate receptors. Rather than simply blocking signalling pathways, these viral mimics actively redirect the host ubiquitination system by recruiting cellular ligases to target antiviral proteins for degradation. In several cases, this strategy is enabled by viral proteins that imitate adaptor motifs used in SCF (Skp1–Cullin–F-box) ubiquitin ligase complexes, reflecting evolutionary pressure to preserve key interaction interfaces within host ubiquitin networks (52).

**Figure 1:**
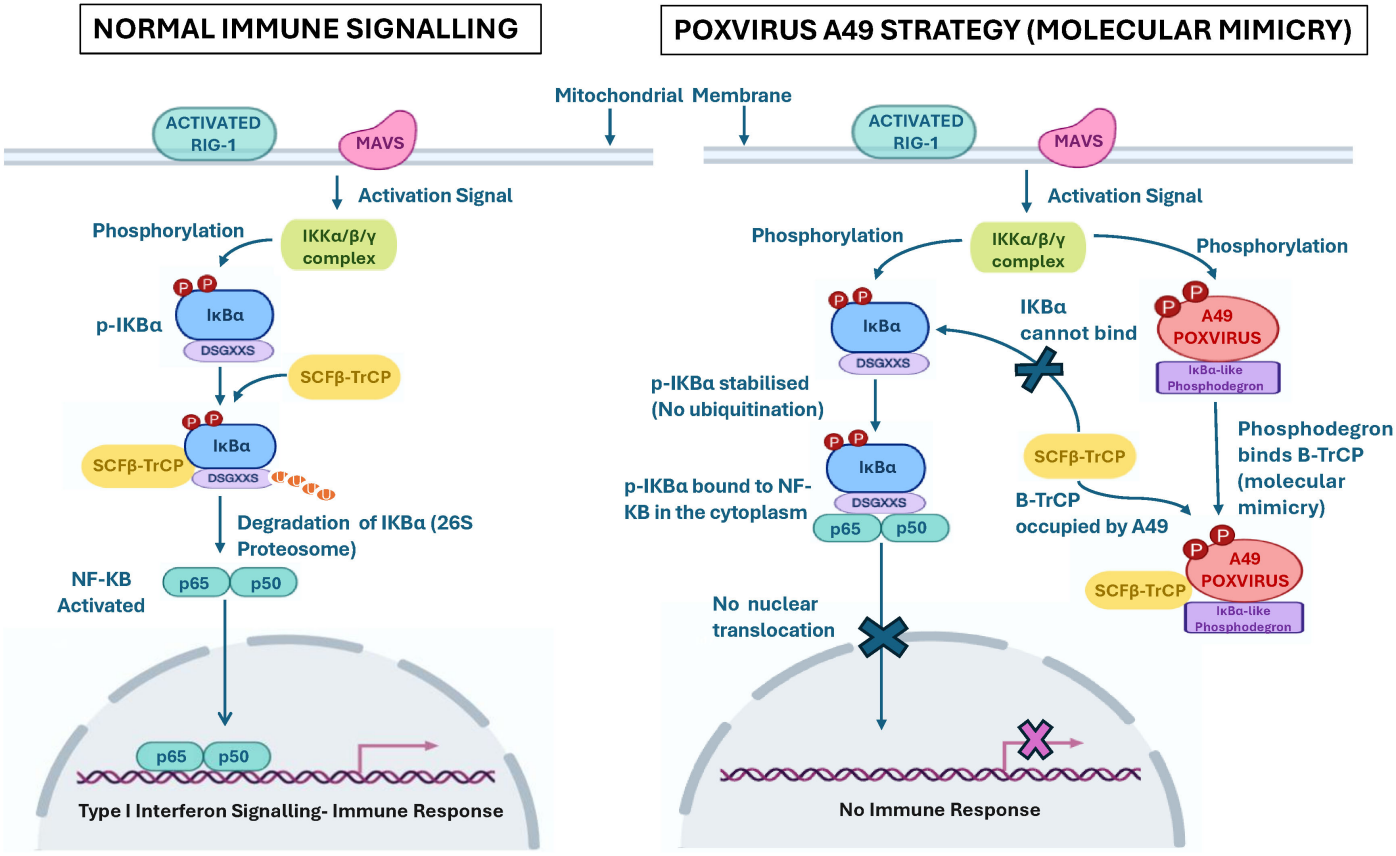
Poxvirus A49 suppresses NF-κB–mediated antiviral signalling by mimicking a host phosphodegron. During normal antiviral signalling, viral RNA recognition activates the RIG-I/MAVS pathway, which stimulates the IKK complex to phosphorylate IκBα. This phosphorylation allows the SCF^β-TrCP ubiquitin ligase to recognize IκBα, leading to its ubiquitination and degradation by the proteasome. The removal of IκBα releases NF-κB, enabling its movement into the nucleus to activate antiviral and inflammatory genes. In contrast, the poxvirus protein A49 contains a similar phosphodegron motif that is phosphorylated by the same host kinase. By binding to β-TrCP as a molecular mimic of IκBα, A49 prevents β-TrCP from targeting endogenous IκBα for degradation. As a result, IκBα remains associated with NF-κB in the cytoplasm, preventing NF-κB nuclear translocation and weakening the host antiviral immune response.

Viruses often use Short Linear Motifs (SLiMs), small 3–10 amino-acid sequences, to mimic host protein interaction sites. Because viral genomes are limited in size, SLiMs allow viruses to manipulate large host protein networks, particularly the ubiquitin–proteasome system, with minimal genetic cost (53). A good example is the A49 poxvirus, which has an IκBα-like SLiM (DSGABS motif) that competes for binding to the SCFβ-TrCP ubiquitin ligase complex. Under normal conditions, β-TrCP ubiquitinates phosphorylated IκBα, leading to its degradation and allowing NF-κB activation. By mimicking this recognition motif, A49 sequesters β-TrCP and stabilizes phosphorylated IκBα, stopping p65 nuclear translocation and dampening type I interferon signalling downstream of RIG-I/MDA5 pathways (Sections 2.1–2.2) (54). The contrast between normal immune signalling and the poxvirus evasion mechanism is illuminated in **Figure 1**. It is observed that while poxviruses suppress immunity by competing for substrates within existing SCF complexes, adenoviruses take a more direct approach by assembling new Cullin-RING ligase (CRL) complexes to redirect ubiquitination toward antiviral host proteins.

The viral proteins E1B-55K and E4orf6 assemble a Cullin-based E3 ubiquitin ligase by recruiting host factors such as Cullin-5 and Elongin B/C. E4orf6 contains motifs that functionally mimic host adaptor proteins, enabling recruitment of these ligase components. Within this complex, E1B-55K binds antiviral substrates including the tumour suppressor p53, directing their ubiquitination and proteasomal degradation, thereby suppressing apoptosis and facilitating viral replication (55).

Unlike adenoviruses, which promote degradation of antiviral proteins, herpesviruses often adopt strategies that preserve host cell survival to support long-term latency. In Epstein-Barr virus (EBV), the latent membrane protein LMP1 functions as a constitutively active mimic of the host CD40 receptor, continuously recruiting TRAF adaptor proteins and activating NF-κB–mediated survival signalling that prevents apoptosis (56). In addition, EBV latent proteins exhibit increased mimicry of host thymic antigens, which may contribute to reduced T-cell recognition and enable lifelong viral persistence (57).

Collectively, these molecular mimicry strategies disrupt ubiquitin-mediated PRR signalling, enabling viruses to evade innate immune detection and establish persistent infections. Historically, targeting these transient host-pathogen interfaces was considered “undruggable.” Because viral motifs closely resemble host protein architectures, therapeutic inhibition carries a significant risk of off-target toxicity and autoimmune cross-reactivity (58). However, recent advances in high-resolution structural mapping have revealed subtle differences among these nearly identical interfaces. Using these datasets, AI-driven modelling can now identify discriminatory features, enabling selective targeting of viral mimicry, from *de novo* inhibitor design to precision protein degradation, as explored in the next section.

## 3 AI-enhanced therapeutics: rewiring the ubiquitin system

### 3.1 The “black box” era vs. structural enlightenment

Prior to AI integration, predictions of post-translational modifications required experimental mapping, mainly analysed short sequence motifs and were built to consider only one PTM at a time (59). AI models fill this gap by using proteome-wide context, learning directly from sequence databases and capturing PTM crosstalk (60). Early deep learning tools for PTM prediction, such as DeepUbiquitin and UbiNet, revolutionized site identification through sequence-based probabilistic models. These convolutional neural network (CNN) and multi-layer perceptron (MLP) frameworks achieved solid predictive performance, with area under the curve (AUC) values of ~0.69 for UbiNets and 0.90 for DeepUbi. These models made use of amino acid motifs and physicochemical properties. Though these models were highly accurate, they functioned as “black box models”, yielding final outputs or predictions (like an 85% probability or an AUC score of 0.90) without elucidating the underlying mechanisms and internal logic used to arrive at that conclusion (61, 62).

Understanding viral evasion requires more than just identifying target residues. It requires the proper visualization of PPIs where actual interactions occur. Sequence-only models like DeepUbiquitin and UbiNet (e.g., 544-D physicochemical vectors in UbiNet) overlook the spatial constraints at E3 ligase interfaces (63). Since they are trained predominantly on human proteomics data, they exhibit false positives in low-homology viral proteins. They also fail to generalize to pathogen-specific strategies for evading ubiquitination. This can be demonstrated by the fact that evolutionary approaches like ESA-UbiSite, which have boosted test accuracy from 0.75 to 0.92 through effective negative screening, remain confined to sequence features and are not able to reveal the 3D RING domain engagement critical for viral mimicry. Hence, researchers are unable to get mechanistic insights for immunology and gene therapy applications (64).

Advanced structural AI tools have overcome the limitations of sequence-only models by providing high-confidence, atomic-level 3D predictions of protein-protein interfaces (PPIs). AlphaFold 3 uses a new diffusion-based approach to accurately predict complex assemblies that include proteins, nucleic acids, and post-translationally modified residues together (65). Independent studies confirm its strong performance in predicting transient antigen-antibody interactions (66). For PTMs specifically, researchers have recently solved a major AI limitation, which is AlphaFold’s inherent inability to model covalent linkages between separate protein chains. Now, modelling of complex polyubiquitin chains is possible through AI-based recreation of isopeptide bonds. This approach overcomes the weak coevolutionary signals that previously made 3D ubiquitination mapping quite difficult (67). Along with these targeted predictions, tools like RoseTTAFold2-Lite can now screen millions of protein pairs to map proteome-wide interactomes. These models confidently identify new structural complexes involving key virulence factors, helping us understand the exact molecular interfaces that human pathogens use to hijack host cellular machinery during infection (68).

Together, these advances have created a wide array of AI tools that operate at different levels, where some predict where ubiquitin attaches, while others predict which E3 ligases mediate those events. Some even model full 3D complexes or generate new inhibitor candidates. To make these roles explicit, we group representative methods in **Table 2** along a few conceptual axes: what they predict, how they represent proteins (sequence vs structure), and whether they generate or score candidates.

**Table 2:** Comparative analysis of AI-driven frameworks in ubiquitin research.

The comparison	Models compared	The key difference	References
Prediction Focus: Site vs E3-substrate Network Prediction	Model 1: UbPred/ESA-UbiSite	UbPred and ESA predict where the ubiquitin attaches (which lysine sites in a protein sequence are ubiquitinated)	(64, 144)
	Model 2: UbiBrowser 2.0	UbiBrowser predicts which host E3 ligase-substrate interaction networks (which ligase acts on which substrate)	(145)
Model Type: Sequence vs. Structure	Model 1: DeepUbi	Sequence-based models that make predictions of ubiquitinated lysine sites	(61)
	Model 2: AlphaFold/HADDOCK	Structural models make predictions based on 3D spatial geometry, revealing specific binding pockets and spatial constraints. (structure/complex predictors that can illuminate ubiquitin interfaces but do not directly output ubiquitination sites)	(146, 147)
Scale: Targeted Accuracy vs. High-Throughput	Model 1: AlphaFold 3	AlphaFold 3 is incredibly accurate for modelling a single, specific viral-host complex	(67)
	Model 2: RoseTTAFold2-PPI	RoseTTAFold2-Lite is lightweight enough to screen millions of protein pairs to discover entirely new interactions	(148)
Design Role: Generative vs. Predictive	Model 1: GANs & RNNs (like LSTMs)	Generative models build entirely new drug molecules based on trained motifs	(149)
	Model 2: MLPs	Predictive models evaluate and score those generated molecules for viability (e.g., antiviral activity)	(150)
Targeting Complexity: Monomer vs. Complex	Model 1: AlphaFold 2	Older models predicted the fold of a single protein	(7)
	Model 2: DegradeMaster	Advanced frameworks like DegradeMaster specifically model the geometry of the entire ternary complex (POI + PROTAC + Ligase)	(92)

This table contrasts key dimensions such as prediction focus (site-specific vs. E3-substrate networks), model type (sequence- vs. structure-based), scale (targeted accuracy vs. high-throughput screening), design role (generative vs. predictive), and targeting complexity (monomer vs. ternary complexes).

Ultimately, the transition from sequence-based “black box” models to advanced structural AI marks a huge leap in our ability to decode antiviral immunity. This structural enlightenment helps us map precise 3D architectures of viral-host interactomes and complex poly-ubiquitin networks. These tools reveal the physical and mechanical aspects of viral infection that cannot be derived from linear sequences. With the clear visualization of specific binding pockets and host-pathogen interfaces, the field is now moving quickly from simply mapping these interactions to actively disrupting them. This is being achieved through the artificial intelligence-driven design of *de novo* inhibitors.

### 3.2 Designing “*de novo*” viral inhibitors

Traditionally, antiviral drug molecules are designed from known datasets and sequences, based on known ligands and active sites or by modifying existing drug molecules. *De novo* sequencing refers to the creation of novel molecules “from scratch” for a specific target or binding interface (69). In recent years, the use of artificial intelligence and generative deep learning models (specifically artificial neural networks) to facilitate the design of *de novo* inhibitors has increased significantly (8, 70). Early methods of design were largely heuristic and required extensive rule-setting. Additionally, these methods worked primarily on 1D or 2D representations, with limited integration of information on 3D protein-pockets (71, 72). With AI, *de novo* design has become much faster and models like DeepLigBuilder can generate chemically and conformationally valid 3D molecules inside a target binding site at a much faster rate (73). Deep learning approaches pose an important advantage over ML approaches, because they use non-linear functions to integrate data at multiple levels, making predictions much more precise (74). DL models can analyse complex, large datasets with multiple features quickly and efficiently. They then use this information to identify active antiviral agents based on their structure, predict drug docking sites on host-viral interfaces, and find new viral proteins to target.

Since *de novo* design involves the creation of new molecules, generative deep learning models are extremely useful in designing drug molecules with specific properties or for specific targets (75). Among these, Artificial Neural Networks (ANNs) are highly favoured for a variety of reasons, but the most significant one is that ANN architecture allows end-to-end frameworks, with one model acting as a generator (for new molecules) while another acts as a predictor to validate the generated sequence by scoring (76, 77). Unlike other model families ANNs can both learn complicated structure-activity relationships and generate new molecules even under multiple design constraints (78). Three types of ANNs are popularly used in *de novo* design: Recurrent Neural Networks (RNNs), Generative Adversarial Networks (GANs), and Multi-Layer Perceptrons (MLPs). The first two are generative models that produce drug molecule candidates, while the latter is predictive and scores each candidate for specific properties (such as those for better antiviral activity) (79).

Within the last decade, many inhibitor candidates for SARS-CoV-2 have been designed using DL-based *de novo* design (80). used a modified LSTM network to generate new molecules, docked the new protein using AutoDock Vina to check its interactions with viral ligands. LSTMs are Long Short-Term Memory networks (a type of RNN) that are trained on labelled data on features of protein structure (hydrophobicity, active sites, disulfide bonds etc) then “seeded” with a short motif to generate an entire new sequence from it (81). These models are typically biased towards antiviral sequences by training them on data containing information on specific ligands, docking sites and inhibitory sequences (82).

While AI-driven design of *de novo* viral inhibitors is a step in the right direction, it still poses the classic challenge with predictions from generative AI models; the patterns recognised are often implicit and unexplained. This implies that the newly designed molecule could potentially be off-target, and there is no foolproof method to validate its structure (83). In other words, while AI has significantly improved the ability to explore chemical spaces, models cannot fully guarantee whether a predicted molecule is active, synthesizable, and developable (84). Alternatively, therapeutics [such as PROTACs (Proteolysis-Targeting Chimeras), AUTACs (Autophagy-Targeting Chimeras), and DUBTACs (Deubiquitinase-Targeting Chimeras)] that strengthen host defence mechanisms against viruses are becoming increasingly popular. Of these, PROTACs are the oldest and most advanced form of targeted degradation technology (85).

### 3.3 Targeted degradation: PROTACs

**Figure 2:**
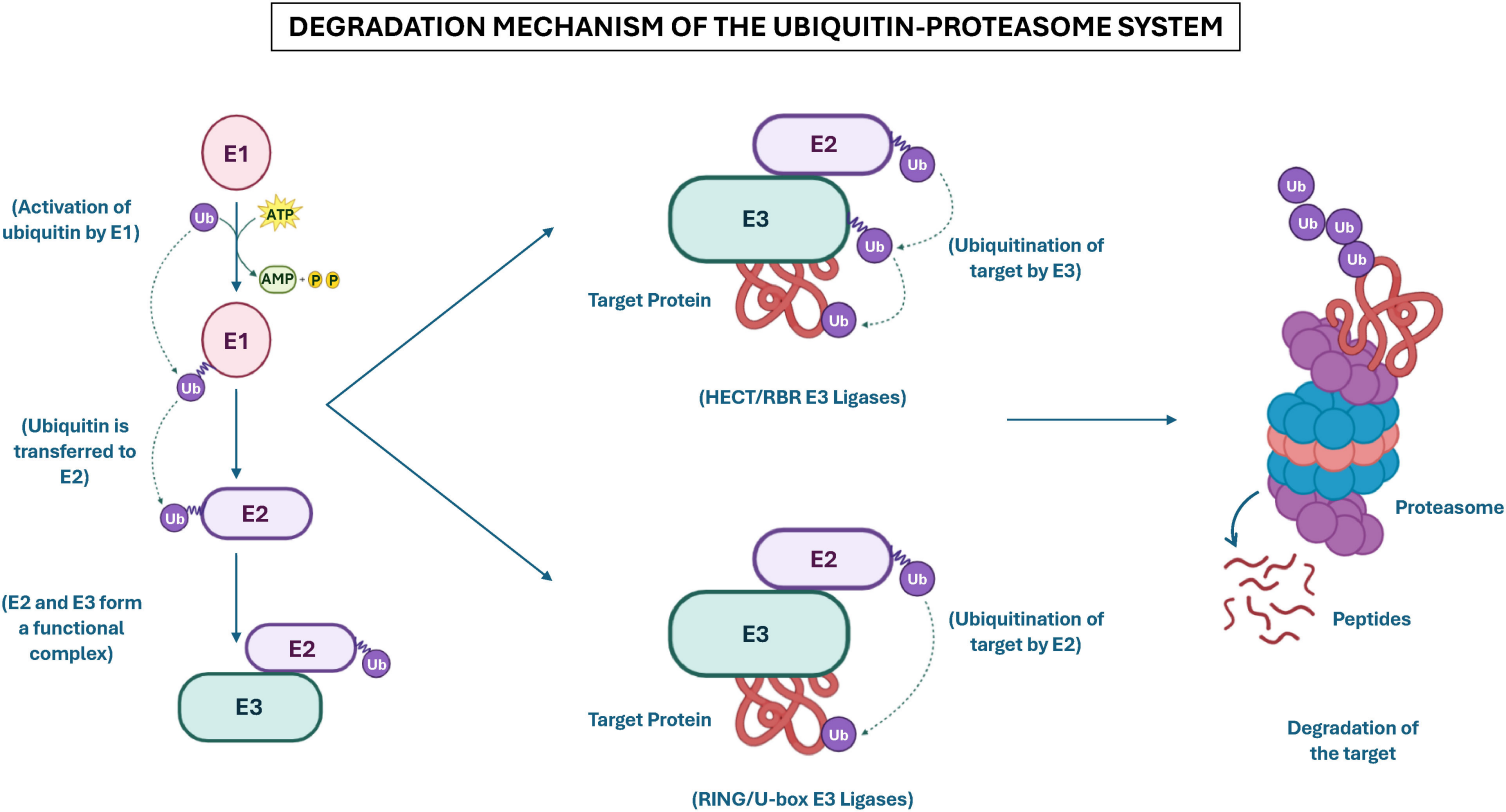
Mechanism for proteasomal degradation via the UPS system. The UPS system is one example of an innate degradative mechanism which breaks down misfolded or damaged proteins, regulatory proteins (for timely regulation of cell signalling) and pathogenic proteins. E1 (ubiquitin activating enzyme) binds ATP and ubiquitin to form a high-energy thioester bond between E1 and ubiquitin while releasing AMP. The activated ubiquitin-E1 complex then transfers the ubiquitin to E2 (ubiquitin conjugating enzyme) which goes on to form a complex with an E3 ligase. This complex then ubiquitinates a target protein, tagging it for degradation by the 26S proteasome. The mechanism of ubiquitination varies across the four broad categories of E3 ligases; RING, HECT, RBR and U-box. For HECT and RBR E3 ligases, E2 must pass the ubiquitin onto E3 before ubiquitination. On the contrary, RING and U-box E3 ligases don’t require this transfer, the E2 complexing with them ubiquitinates the target directly.

In contrast to the occupancy-driven inhibitors which block viral active sites (refer to section 3.2), targeted protein degradation (TPD) leverages the host’s ubiquitin-proteasome system (UPS) against viral hijackers. The underlying mechanism relies on a sequential enzymatic cascade (refer to **Figure 2**). The process is driven by 3 distinct classes of enzymes: ubiquitin-activating enzymes (E1), ubiquitin-conjugating enzymes (E2), and ubiquitin ligases (E3). Ubiquitin is activated by E1 in an ATP-dependent process, transferred to E2 via trans-thioesterification, and then passed to the target protein by E3 ligases, leading to polyubiquitination. When these ubiquitin monomers are linked specifically through their K48 residues, they act as signals for the proteolytic degradation of the protein into short peptide fragments by the 26S proteosome. Recent studies further emphasize that the UPS and PROTAC-mediated degradation represent promising therapeutic avenues in cancer and infectious diseases (86, 87). Proteolysis Targeting Chimeras (PROTACs) are small tripartite molecules consisting of a ligand for the target protein of interest (POI), a ligand for the E3 ligase, and a linker. A PROTAC only needs to transiently bind the viral POI to bring the E3 ligase in proximity to it, thereby tagging the viral target for polyubiquitination. This leads to the subsequent degradation of the viral protein by the host UPS. PROTACs are used in dismantling pathogens and targeting previously ‘undruggable’ proteins (88, 89).

The efficiency of targeted protein degradation (TPD) depends heavily on the linker geometry. Suboptimal angles or lengths cause clashes due to steric hindrance, aborting degradation. Tackling this linker bottleneck calls for computational solutions, especially rational AI-driven design (90). Deep neural networks such as DeepPROTACs now use Graph Convolutional Networks (GCNs) to analyse the 3D binding pockets of both the target and the E3 ligase, combined with sequence-based AI, to evaluate the linker’s chemistry. This predicts the actual degradation capacity with high accuracy before synthesis (91). Meanwhile, frameworks like DegradeMaster utilize E(3) equivariant 3D graph neural networks to model the precise spatial geometry of the entire ternary complex (POI-PROTAC-Ligase) (92). Additionally, interpretable ML models like PrePROTAC evaluate genome-wide target proteins and their susceptibility to E3 ligase degradation before they even enter a lab (93).

While these AI models optimize the structural design, the experimental validation of the predicted linkers is often slowed by the challenges associated with purifying complex molecules. To speed up this process, miniaturized Direct-to-Biology (D2B) platforms now utilize high-efficiency “click chemistry” to synthesize PROTAC libraries, such as 92 crude soluble epoxide hydrolase (sEH) degraders (94). However, skipping the purification step poses a challenge, which is to determine if the low biological activity is due to weak molecular potency or poor chemical yield. ML-based deconvolution frameworks resolve this by using Bayesian models to isolate these variables. This framework was validated against the SARS-CoV-2 main protease (Mpro), where it successfully identified nanomolar inhibitors previously masked by low yields (95).

Although PROTACs are considered promising degradative drugs because they can selectively target and degrade viral proteins through the UPS, their antiviral potential still requires further investigation due to several unresolved challenges. These include the risk of off-target degradation of host cellular proteins, possible toxicity, poor pharmacokinetic properties such as limited bioavailability and tissue penetration caused by their large molecular size. There are also uncertainties regarding long-term safety and effects on viral evolution. In addition, more preclinical and clinical studies are needed to evaluate their efficacy, optimize delivery systems, and address ethical and regulatory concerns before PROTACs can be widely applied as antiviral therapies (96).

Building on the concept of targeted protein degradation by PROTACs, researchers have also explored alternative degrader technologies with simpler structures and potentially improved pharmacological properties. One such emerging approach is molecular glue degraders, which promote or stabilize interactions between target proteins and E3 ubiquitin ligases, leading to selective protein degradation. These molecules represent a promising next generation of targeted degradation strategies in antiviral therapy.

### 3.4 Reprogramming “undruggable” interfaces with molecular glue degraders

Much like PROTACs, molecular glues leverage the host’s UPS system as well, except they don’t require a linker molecule, and they typically don’t bind the viral target that is meant to undergo degradation. They are designed to disrupt the protein-protein interface (PPI) between a host E3 ligase and a viral protein (which binds to the host E3 to block its ubiquitinating ability), causing a conformational change that restores the function of the E3 ligase. Once the host E3 has successfully tagged the viral target with ubiquitin, the 26S proteasome detects and degrades it. **Figure 2** shows the underlying innate mechanism for proteasomal degradation via the UPS system (97).

Transcription factors (TFs) and viral-host PPIs between viral proteins and host E3 ligases are common targets for antiviral therapy. However, these proteins and interfaces have a shallow, extended topology that doesn’t provide a clear docking site for typical small molecule drugs, making them “undruggable” (98). PPI interfaces function like Velcro straps; they are wide and largely flat with small chemical interactions spanning the interface, resulting in the combined effect of tethering the two proteins together (99). This topology does not have deep obvious grooves with a small surface area to utilize as targets for typical small-molecule drugs. Instead, the entire large interface has polar sidechains and hydrophobic contacts, creating a structure that lacks the depth to anchor PPI modulators (less than 0.01% are effective), even though they are built larger to improve bioactivity (100).

**Figure 3:**
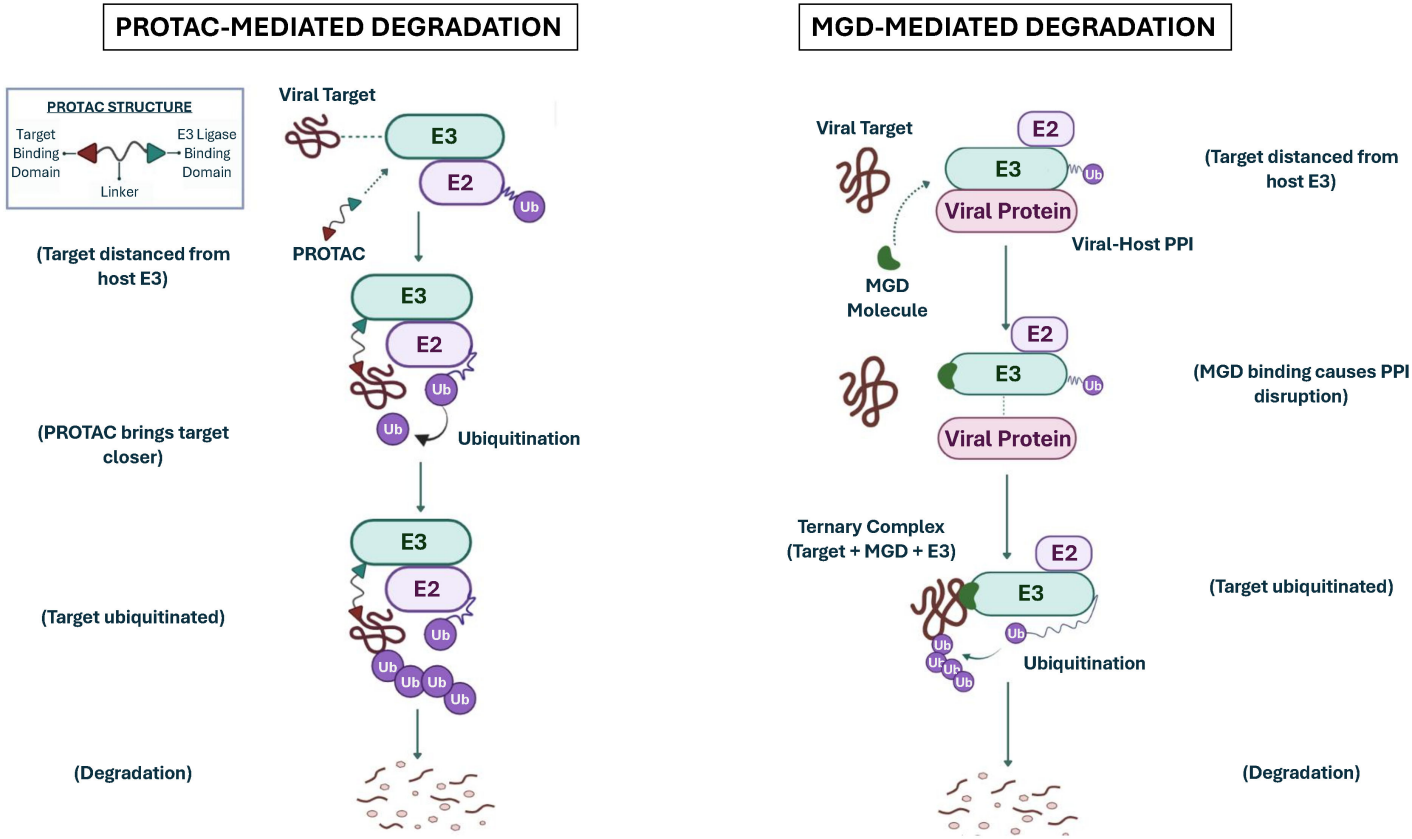
Mechanistic comparison of PROTAC- and MGD-mediated targeted protein degradation. PROTACs are heterobifunctional molecules composed of two binding domains connected by a linker, enabling simultaneous interaction with a target protein and an E3 ubiquitin ligase. This induced proximity promotes polyubiquitination of the target, marking it for degradation by the proteasome. In contrast, molecular glue degraders (MGDs) are small molecules that bind at a protein–protein interface and induce a conformational change, stabilizing or redirecting interactions between a host E3 ligase and a new substrate. This reprogramming enables the ligase to recognize and ubiquitinate the target protein, leading to its subsequent proteasomal degradation.

Molecular degraders that weaponize the host’s ubiquitin-proteasome system, to destroy the viral-host PPI, are being developed as the solution to drugging these “undruggable” interfaces (101). Previously explained PROTACs follow this approach to induce proteasomal degradation, however Molecular Glue Degraders (MGDs) can induce degradation without needing an additional linker (102). **Figure 3** showcases the resultant differences in their degradative mechanisms. MGDs attach to host factors that viral proteins are dependent on (usually E3 ligases) and cause a conformational change which changes the binding target (substrate) for the host factor. This new ternary complex, enables the UPS system in two ways: (a) the host factor binds to a free viral protein and ubiquitinates it, tagging it for degradation, rendering the virus unable to replicate; (b) by tagging a bound host factor for degradation, which would remove the viral protein’s PPI, leaving it free for targeted proteolysis (97). This ability to exploit a protein’s shape complementarity and turn weak and nonproductive interactions into productive docking sites for induced degradation has made MGDs attractive for viral proteins with scaffolding based functions (103).

Design of MGDs requires structural information on specific binding sites outside the viral-host PPI, however most existing databases mainly contain docking sites for larger conventional small molecule drugs. This is where predictive DL models are becoming increasingly useful. J. Wang et al. developed a structure-based generative DL model, GENiPPI to map PPIs using a Convolutional Neural Network (CNNs) and an LSTM module to study latent relationships between PPIs and active compounds to suggest new drug-like molecules to disrupt the PPI scaffold (104).

MGDs hold significant promise for antiviral therapeutics due to their various advantages over traditional inhibitors. Unlike inhibitors that simply block protein function, MGDs could catalytically eliminate viral proteins entirely, prolonging pharmacodynamic effects since protein function would return only after new protein synthesis (105). Additionally, MGDs may be less susceptible to resistance mutations because their activity depends on ternary complex stability rather than binding affinity alone, meaning loss of the original binding interaction does not always destroy the function (106). However, MGDs are difficult to design rationally, and existing degraders were discovered serendipitously, meaning the field remains at proof-of-concept stage with no validated antiviral MGDs (107). Thus, MGDs represent a significant research opportunity where PROTACs have demonstrated initial promise but molecular glues offer superior clinical potential.

**Table 3** compares the AI application and antiviral capability of the strategies discussed in this review alongside some other antiviral therapies which have produced clinically available results.

**Table 3:** Critical analysis of AI-enabled antiviral therapeutic modalities.

Therapeutic type	AI framework	Antiviral potential & applications	Limitations	References
*De novo* antiviral inhibitors	RNNs read and learn chemical syntax data of various molecular structures to generate non-peptide inhibitor molecules with antiviral properties. Virtual screening and structure-based refinement are used to select the optimal inhibitor. Advanced models (like Chemistry42) usually combine RNNs with other algorithms rather than rely on a single model.	Can enforce multi-parameter objectives and rapidly generate many target-specific scaffolds, exploring chemical spaces beyond known antivirals.	Many designs are target-specific and have limited benchmarking on non-Mpro targets and/or emergent variants. Long-term stability against viral resistance and fitness costs remains untested.	(151, 152)
		Example: ISM3312 Targets SARS-CoV-2 Mpro (highly conserved across strains and key to the viral life cycle) and shows incredible antiviral activity across strains with low off-target risk. It has undergone extensive preclinical characterisation, but there is no clinical efficacy data yet.		
PROTACs	ML models (such as DeepPROTACs) predict degradation efficiency and prioritize linker-warhead combinations. Other models (PROTAC-specific PK predictors) support optimization of properties such as clearance and synthetic tractability.	Unlike typical antiviral peptides or inhibitors, PROTACs degrade the target protein rather than just block its function, meaning protein function is harder to restore. This makes them likely to have a more prolonged antiviral effect.	Existing models are yet to be used to design antiviral PROTACs because they are all trained on oncology datasets with limited generalizability to viral targets. PROTACs are large molecules with complex structures limiting their bioavailability and manufacturability.	(91, 153)
		Example: V3 (designed traditionally with CADD, not AI) Induces robust degradation of HA and shows broad-spectrum anti-influenza A activity across multiple strains. V3 demonstrates antiviral activity in mouse models but remains at a proof-of-concept stage with no information on human pharmacokinetics.		
Molecular Glue Degraders	There are few AI-based prediction models for MGDs however, cheminformatics and ML models are used to mine chemotype–E3 pairs and prioritize glue-like scaffolds. MOLDE, is a recent model which uses established MGDs to reproduce known binding modes and evaluate new input structures based on thermodynamic favourability, stability etc	Like PROTACs, MGDs catalytically eliminate target proteins, rather than just block them. Additionally, their activity does not depend on binding affinity, making them less susceptible (but not immune) to viral resistance.	MGD discovery is largely serendipitous since they are difficult to design. Current predictive models are early-stage and trained mostly on oncology glues. Viral/host sequence variation can disrupt glue binding epitopes, if there is no cooperative ternary complex formation.	(106, 154, 155)
Antiviral Peptides (AVPs)	Generative models (GANs, VAEs, RL) are used to propose novel sequences with predicted antiviral properties using databases (such as AVPdb) to train them on existing high activity peptides. Structure−based docking and MD simulations are then used for target−specific refinement.	AI models allow rapid exploration of sequence space and designed peptides can be tuned and optimized for high specificity and desired antiviral activity.	Most AI-based designs are supported only by in silico or *in vitro* data, lacking experimental validation. Many existing clinical AVPs were discovered pre-AI. AVPs can have poor oral bioavailability or be immunogenic; constraints which AI models don’t usually account for.	(156, 157)
		Example: SARS-CoV-2 PEP 49 Binds to the SARS-CoV-2 Spike protein; a structural protein that facilitates viral entry into host cells Still at the conceptual/preclinical stage with *in silico* supporting data.		
Antiviral drug repurposing	AI and network-medicine frameworks integrate clinical data and drug–target networks to prioritize existing drugs and combinations. Then, DL models rank drugs based on predicted antiviral efficacy and categorize the patient subgroups most likely to benefit.	Drug repurposing significantly mitigates time constraints imposed by rapid viral evolution and resistance and reduces safety risks by focusing on drugs with known clinical profiles.	Repurposing requires ML models to be trained on small, specific datasets, meaning AI-prioritized drugs may not be generalizable. These drugs often modulate host signalling pathways, which can cause toxicity or immunosuppression, and AI models struggle to predict this context-driven performance Clinical trials of AI-prioritized repurposing candidates have shown mixed or modest efficacy.	(158, 159)
		Example: Remdesivir (originally developed for Ebola virus) Targets RNA−dependent RNA polymerase (RdRp), thereby inhibiting SARS−CoV−2 replication. It was the first approved antiviral for COVID−19.		

This table summarizes how AI is applied in the design of small molecule inhibitors, targeted protein degraders, antiviral peptides and repurposed drugs and compares the antiviral potential, limitations and clinical availability of each AI-enhanced therapeutic strategy.

## 4 Challenges and future directions of AI-driven antiviral therapeutics

### 4.1 Bridging the interpretability gap in AI-driven biology

On one hand, artificial intelligence has accelerated key tasks in ubiquitination and antiviral immunity, such as identifying host-virus protein interactions, predicting ubiquitination sites, and designing antiviral targets. On the other hand, it has introduced a major challenge: the “interpretability gap.” While foundational DL models show remarkable accuracy, they often act as black boxes, making it difficult to understand the key decisions underlying the final output (108).

In sensitive fields like healthcare, predictive accuracy alone is not enough to establish trust, especially with patients. Researchers and regulatory bodies require an explicitly transparent understanding of the mechanistic logic driving AI-generated predictions to ensure safety and efficacy. Recent literature emphasizes that moving beyond “explainable AI” to “trustworthy AI” is essential, ensuring that AI systems are supported by robust validation, high-quality data, and stringent ethical, legal, and regulatory safeguards (109, 110).

That said, the push for XAI in healthcare is not universal. It can be argued that XAI may not necessarily provide insights relevant to clinical decision-making and can lead to misplaced trust or misinterpretation. A major flaw of XAI is that it tempts users to confuse correlation with causation. AI finds statistical patterns, not biological causes. Trying to force biological meaning onto XAI outputs can be dangerous. If an AI’s explanation matches what a researcher expects, it creates a false sense of security. On the other hand, if the explanation seems strange or unexpected, researchers might incorrectly reject a highly accurate model. Therefore, it can be argued that in some cases, more than reliance on XAI, rigorous validation and impact studies using black box models may be more reliable and trustworthy (111). Still, interpretability remains crucial where biological mechanisms drive decisions, with recent efforts embedding transparency directly into models.

Recent approaches in structural biology aim to reduce the interpretability gap by combining accurate predictions with biological features such as protein structure, sequence, and physicochemical properties. For instance, InteracTor uses explainability methods to highlight which features drive their predictions. This allows researchers to connect AI outputs to real biological mechanisms, which improves both understanding and trust in these systems (112). By using explainability techniques such as integrated gradients to probe both input sequences and internal layers, researchers can now identify the specific amino acids and model components driving predictions, revealing that transformer models focus on biologically meaningful regions like active and binding sites (113). Identifying such key residues may help pinpoint interaction motifs involved in host–virus protein interactions and immune regulation.

Similarly, GPS-DTI integrates graph neural networks, protein language models, and cross-attention mechanisms to accurately predict drug–target interactions while highlighting key molecular interaction regions, thereby combining strong generalization with interpretable, biologically meaningful insights (114).

Designing AI-enhanced therapeutics such as PROTACs also requires interpretable ML frameworks. For example, PROTAC-STAN, a structure-informed DL framework, addresses black-box limitations by employing a ternary attention mechanism that models target–PROTAC-E3 ligase interactions. It improves prediction accuracy while ensuring mechanistic interpretability, making it easier to design effective targeted protein degradation therapies (115).

To make these approaches more practical, AI models should ideally combine explainability with rigorous external validation, human-in-the-loop review, and mechanistic experimental testing, so that model outputs are not only interpretable but also biologically reliable (116).

Although explainability strengthens trust in AI outputs, the broader challenge is whether these predictions can be converted into therapeutically viable candidates.

### 4.2 Translational and clinical applicability

The translational and clinical applicability of AI-driven ubiquitin-targeted antiviral therapeutics remains promising but is still in its early stages of development. Therapeutic strategies such as AI-designed PROTACs, molecular glues, and *de novo* inhibitors offer innovative approaches to disrupt viral replication through UPS manipulation, as shown in previous sections. Although these approaches show strong mechanistic and preclinical potential, antiviral PROTACs remain largely limited to preclinical *in vitro* degradation assays and animal infection models. Importantly, several TPD therapeutics, including PROTACs such as ARV-471 and BGB-16673 for cancer, KT-474 for autoimmune diseases, and many more, have already entered clinical trials, providing precedents for future antiviral applications (117, 118).

Key translational challenges in AI-driven antiviral discovery include limited high-quality datasets, inconsistent assay protocols, poor model generalisability across datasets, limited interpretability of black-box models, and poor synthetic accessibility for the manufacturing of many AI-generated compounds. Incompatible datasets and limited experimental feedback also reduce the clinical reliability of AI predictions. Addressing these challenges will require robust experimental validation, standardised protocols, multimodal AI systems integrating structural and biological datasets, and XAI frameworks to improve predictive accuracy and mechanistic transparency. Regulatory translation is also complicated by requirements for audit trails, GxP compliance, and interpretable mechanistic rationale once AI-derived predictions influence preclinical or clinical decision-making (6, 119).

PROTACs, for instance, have several pharmacokinetic constraints, such as bioavailability and tissue penetration difficulties (96). Nanotechnology-based delivery systems may help overcome these barriers by improving stability, tissue targeting, and intracellular delivery of AI-designed antiviral therapeutics while reducing systemic toxicity. The clinical success of lipid nanoparticle-based mRNA vaccines highlights the therapeutic potential of nanotechnology in antiviral medicine. For example, nanozymes such as Ag-TiO2 single-atom nanozymes have demonstrated potent anti-SARS-CoV-2 activity by enhancing macrophage-mediated viral clearance and lysosomal antiviral effects (6).

Beyond these macroscopic translational challenges and their potential solutions, there remains a critical bottleneck underlying the deployment of AI itself for therapeutic applications, which is its propensity to hallucinate.

### 4.3 Hallucinations vs reality: reliability of AI-generated predictions

A major risk of using AI models is their tendency to “hallucinate”, producing entirely fabricated or biologically incorrect outputs that may still appear highly credible. In the context of PTMs, an AI model might predict plausible but non-existent ubiquitination sites or invent PPIs within antiviral pathways. These errors are due to the models’ underlying design, which optimizes for coherent language and data patterns rather than verified scientific accuracy (15).

It is important to understand the reason behind these fabrications to develop more robust predictive tools. Recent frameworks categorize hallucinations into two primary types: prompt-induced and model-intrinsic. Prompt-induced hallucinations occur when the input prompt is unclear, vague, or incorrect. In this paper’s context, these hallucinations may occur when queries about specific ubiquitin ligases or immune signalling cascades are vague or incorrect, leading the model to generate ungrounded responses. Prompt-induced hallucinations can be partially reduced by prompt-tuning strategies such as Chain-of-Thought prompting and Self-Consistency, which encourage more robust reasoning without altering model parameters. In contrast, model-intrinsic hallucinations reflect deeper structural limitations, where the probabilistic model incorrectly favours a hallucinatory output over a factual one because of how it has been trained (120). For example, it is possible that models mapping ubiquitination sites might prefer to give definitive answers, rarely acknowledging knowledge gaps, even when the specific viral-host interaction data is lacking.

To address these model-level limitations, one strategy is the deployment of Retrieval-Augmented Generation (RAG) coupled with structured Knowledge Graphs (KGs). Rather than relying solely on a model’s internal probabilistic memory, these architectures force the AI to dynamically ground its predictions in verified databases before generating an output (121).

In practical terms, hallucinations can be reduced by using knowledge graphs, multi-stage training, expert feedback, and strong evaluation through testing and real-world validation before predictions are used in antiviral or ubiquitination research (122).

Beyond ensuring factual reliability, an equally important challenge is capturing the dynamic nature of protein interactions, which often occur in changing structural and functional states rather than as fixed static complexes.

### 4.4 The 4th dimension: modelling dynamic protein interactions

“4D modelling” is the term that has come to describe the integrative approach of combining static 3D structures with time-resolved conformations to capture the dynamic behaviour of protein complexes. In other words, AI models can already predict sites of protein interactions and now we must attempt to predict when and how these interactions occur (123).

Protein complexes (including the UPS system) interact quickly and dynamically, tagging target molecules, assembling and disassembling complexes, and recruiting and detaching regulatory factors. Current AI models for protein structure prediction have limited flexibility, with a tendency to provide static, single-state structures that miss dynamic conformational changes occurring during protein interactions (124). These models used protein databases that contain information on static structures with limited information on conformational diversity, often leading to hallucinatory outputs that may seem accurate, but fail to function under experimental conditions (65). To resolve this, next-generation AI can be trained to learn the intermediate dynamic forms of protein complexes (ligand-bound, post-translationally modified, etc.) to make it structure-aware, time aware and physics-informed (125).

Implementation of this 4D-style framework requires datasets which contain the structures of intermediate states of these complexes. Time-resolved cryo-electron microscopy (cryo-EM) is an emerging, powerful tool which freezes protein complexes at different times during an interaction, producing visualisations of intermediate, transient states (126). Zhang et al. (127) used time-resolved cryo-EM to visualise transition states of the human proteasome to learn the impact of the deubiquitinating enzyme USP14 on its degradative activity. It’s a valuable tool which can be integrated with AI based data processing to match, filter and rank intermediates enabling effective identification of therapeutic targets.

In recent years, computational methods have advanced to better model the dynamic and kinetic aspects of protein interactions. Huang et al. (128) developed *Pathfinder*, a computational tool designed to predict protein folding pathways from information on seed states and transition probabilities. They have used Metropolis Monte Carlo (MMC) trajectories to generate conformations, clustering accepted conformations using the *SPICKER* clustering approach (each cluster is a “seed state”) developed by Y. Zhang & Skolnich (129) in 2004. Pathfinder then uses energy functions to estimate transition probabilities, capturing the states most likely to precede or follow others. By tracing this sequence of seed states, it maps the most likely folding pathway of the sample protein.

Similarly, Y. Liu et al. (130) developed TransDSI, a sequence-based deep learning framework to predict deubiquitinase-substrate interactions (DSIs) using transfer learning and an explainable module. They used a graph encoder as a feature embedding module. The encoder is pre-trained on proteome-wide evolutionary relationships (thereby learning biological patterns such as conserved motifs) and creates a numerical matrix of protein embeddings. Then the prediction module, DSI-Predictor, fine-tunes the model to a smaller dataset of validated DSI pairs (the model learns how DSIs manifest), so that when a query sequence of a DUB and substrate is input, it outputs a probability score to indicate the likelihood of interaction. A third module, PairExplainer, highlights critical regions with the highest contribution to DSI prediction, allowing partial mapping of the functional basis of each interaction (DUB or substrate sequence regions that are most crucial). The mapping from TransDSI can be used as a basis for structure-guided drug design. TransDSI can also identify disease-specific DSIs (such as those relevant to cancer), which can be utilised for drug-targeting.

In theory, 4D modelling is a broadly generalizable concept, but the practical applicability is yet to be explored. While both Pathfinder and TransDSI demonstrate a high potential for becoming dynamic structure prediction models, there is currently limited evidence to experimentally validate their disease-specific applications.

Going forward, we need models that can predict functionally relevant transient protein complexes, given that many protein interactions that govern cellular signalling are inherently “4D” (non-static). These models could be trained on kinetic simulations and dynamic intermediate complexes to model the co-evolution of protein interactions (such as host E3 ligases and vDUBs) by dividing them into time steps and learning motion patterns at each stage (131). Eventually, the AI could generate drug molecules with spatio-temporal control that disrupt protein dynamics (such as assembly and disassembly of complexes) as suggested by Wang et al. (132).

Taken together, these advances show that the next stage of AI in this field will require not only better prediction accuracy, but also stronger biological grounding, dynamic modelling, and practical therapeutic translation.

## 5 Conclusion

Post-translational modifications (PTMs) such as ubiquitination, phosphorylation, SUMOylation, and ISGylation are critical for enabling the innate immune system’s response to viral infections. Ubiquitination is often considered one of the most important PTMs in antiviral signalling, as it acts as a versatile regulatory signal that can either activate or inactivate pathways through K63- and K48-linked chains, respectively. K63-linked ubiquitination activates signalling pathways that recruit interferons, while K48-linked ubiquitination marks PRRs for proteasomal degradation. From sections 2.3 & 2.4, it is evident that viruses frequently hijack the ubiquitination system to evade host immune responses, either by mimicking host proteins or by redirecting E3 ligases to destroy host antiviral machinery.

In recent years, there has been a marked increase in the application of AI-driven models in healthcare. This review highlights several emerging AI models that can be applied for structural prediction (such as AlphaFold), generative designing (GANs/RL for inhibitors), and targeted manipulation of the UPS (AI-optimized PROTACs/MGDs). This illustrates how AI-driven therapeutics intersect with ubiquitination, a regulator of antiviral immune responses. At the same time, there are some core challenges that need to be addressed when using AI models, such as black box opacity, hallucinations, and data limitations. By considering these strengths and limitations together, this review reveals how AI can be used to target ubiquitination’s vulnerabilities to counter viral evasion.

AI-driven modelling has begun to address long-standing antiviral challenges, particularly at ubiquitin-regulated interfaces. By rapidly predicting viral–host protein interactions and their conformational changes, these models can help in finding candidates for PROTAC-like strategies against viral proteins that exploit host E3 ligases. Expanding structural datasets from cryo-EM and deep-learning predictors helps reduce bias and problems due to data scarcity, especially if coupled with XAI tools that make predictions about ubiquitin signalling and viral evasion mechanisms more interpretable.

For future advancements in this field, challenges associated with AI models, such as black-box issues, data scarcity, bias in viral-host models, and hallucinations, must be adequately addressed. Black box issues can be addressed through retrieval-grounded and human-in-the-loop AI workflows, data scarcity can be addressed by multi-omics integration, transfer learning, and federated learning, bias in viral–host models can be reduced through diverse datasets and external validation and hallucinations can be reduced through retrieval-grounded, database-linked workflows. Moreover, nanoparticle-based strategies are a promising future direction for delivery, targeting, and antiviral modulation. However, research utilising these concepts in healthcare is limited, and these advances have not yet been systematically unified around ubiquitin-centred antiviral targets, or AI-designed degraders, and explicit ethical and governance models tailored to such therapeutics remain underdeveloped.

Aligning ubiquitin-mediated control of innate antiviral immunity with AI-enhanced therapeutic design may lay the groundwork for a new generation of precise, mechanism-driven antiviral therapeutics.

## References

[1] Mao AP Li S Zhong B Li Y Yan J Li Q . Virus-triggered ubiquitination of TRAF3/6 by cIAP1/2 is essential for induction of interferon-β (IFN-β) and cellular antiviral response. J Biol Chem. (2010) 285:9470–6. doi: 10.1074/jbc.M109.071043 20097753 PMC2843197

[2] Chathuranga K Weerawardhana A Dodantenna N Lee JS . Regulation of antiviral innate immune signaling and viral evasion following viral genome sensing. Exp Mol Med. (2021) 53:1647–68. doi: 10.1038/s12276-021-00691-y 34782737 PMC8592830

[3] Tu D Zhu Z Zhou AY Yun C Lee KE Toms AV . Structure and ubiquitination-dependent activation of TANK-binding kinase 1. Cell Rep. (2013) 3:747–58. doi: 10.1016/j.celrep.2013.01.033 23453972 PMC3863638

[4] von Delft A Hall MD Kwong AD Purcell LA Saikatendu KS Schmitz U . Accelerating antiviral drug discovery: lessons from COVID-19. Nat Rev Drug Discov. (2023) 22:585–603. doi: 10.1038/s41573-023-00692-8 37173515 PMC10176316

[5] Schauperl M Denny RA . AI-based protein structure prediction in drug discovery: impacts and challenges. J Chem Inf Model. (2022) 62:3142–56. doi: 10.1021/ACS.JCIM.2C00026 35727311

[6] Du S Hu X Li P Xu S Kim M Liu X . Antiviral drug discovery and development: challenges and future directions. Signal Transduct Target Ther. (2026) 11:69. doi: 10.1038/S41392-025-02539-7 41735249 PMC12932771

[7] Jumper J Evans R Pritzel A Green T Figurnov M Ronneberger O . Highly accurate protein structure prediction with AlphaFold. Nature. (2021) 596:583–9. doi: 10.1038/s41586-021-03819-2 34265844 PMC8371605

[8] Mashhadi Abolghasem Shirazi M Haghighat S Nikbakht Z Salimkia E Kiumarsy A . Next-generation antiviral peptides: AI-driven design, translational delivery platforms, and future therapeutic directions. Virus Res. (2025) 361:199642. doi: 10.1016/J.VIRUSRES.2025.199642 41106780 PMC12713194

[9] Das P Sercu T Wadhawan K Padhi I Gehrmann S Cipcigan F . Accelerated antimicrobial discovery via deep generative models and molecular dynamics simulations. Nat BioMed Eng. (2021) 5:613–23. doi: 10.1038/s41551-021-00689-x 33707779

[10] Lin CT Shiau YP Lin CC . Machine learning in targeted protein degradation drug design: a technical review of PROTACs and molecular glues. Drug Discov Today. (2026) 31:104563. doi: 10.1016/J.DRUDIS.2025.104563 41318024

[11] Li K Crews CM . PROTACs: past, present and future. Chem Soc Rev. (2022) 51:5214. doi: 10.1039/D2CS00193D 35671157 PMC10237031

[12] Sarker IH . Deep learning: a comprehensive overview on techniques, taxonomy, applications and research directions. SN Comput Sci. (2021) 2:420. doi: 10.1007/S42979-021-00815-1 34426802 PMC8372231

[13] Samal BR Loers JU Vermeirssen V De Preter K . Opportunities and challenges in interpretable deep learning for drug sensitivity prediction of cancer cells. Front Bioinf. (2022) 2:1036963. doi: 10.3389/fbinf.2022.1036963 PMC9714662 36466148

[14] Ornes S . Peering inside the black box of AI. Proc Natl Acad Sci USA. (2023) 120:e2307432120. doi: 10.1073/PNAS.2307432120 37224179 PMC10235959

[15] Rouzrokh P Khosravi B Faghani S Moassefi M Shariatnia MM Rouzrokh P . A current review of generative AI in medicine: core concepts, applications, and current limitations. Curr Rev Musculoskeletal Med. (2025) 18:246–66. doi: 10.1007/S12178-025-09961-Y 40304941 PMC12185825

[16] Pallikkavaliyaveetil N Chandrasekaran S . Small data, big challenges: machine- and deep-learning strategies for data-limited drug discovery. Adv Drug Delivery Rev. (2026) 229:115762. doi: 10.1016/J.ADDR.2025.115762 41421504 PMC13094559

[17] Muralidharan V Adewale BA Huang CJ Nta MT Ademiju PO Pathmarajah P . A scoping review of reporting gaps in FDA-approved AI medical devices. NPJ Digital Med. (2024) 7:273. doi: 10.1038/s41746-024-01270-x 39362934 PMC11450195

[18] Ochsner N Bouman J Vaughan T Stadler T Bonhoeffer S Regoes R . Viral simulation reveals overestimation bias in within-host phylodynamic migration rate estimates under selection. Mol Biol Evol. (2026) 43. doi: 10.1093/MOLBEV/MSAG014 41622429 PMC12911929

[19] Lin D Zhong B . Regulation of cellular innate antiviral signaling by ubiquitin modification. Acta Biochim Biophys Sin (Shanghai). (2015) 47:149. doi: 10.1093/abbs/gmu133 25651846 PMC7109689

[20] Cai C Tang YD Xu G Zheng C . The crosstalk between viral RNA- and DNA-sensing mechanisms. Cell Mol Life Sci. (2021) 78:7427. doi: 10.1007/s00018-021-04001-7 34714359 PMC8554519

[21] Carty M Bowie AG . Recent insights into the role of Toll-like receptors in viral infection. Clin Exp Immunol. (2010) 161:397. doi: 10.1111/j.1365-2249.2010.04196.x 20560984 PMC2962956

[22] Bryant CE . Rethinking Toll-like receptor signalling. Curr Opin Immunol. (2024) 91:102460. doi: 10.1016/j.coi.2024.102460 39288726

[23] Uematsu S Akira S . Toll-like receptors and type I interferons. J Biol Chem. (2007) 282:15319–24. doi: 10.1074/jbc.R700009200 17395581

[24] Chiang JJ Davis ME Gack MU . Regulation of RIG-I-like receptor signaling by host and viral proteins. Cytokine Growth Factor Rev. (2014) 25:491. doi: 10.1016/j.cytogfr.2014.06.005 25023063 PMC7108356

[25] Levy DE Marié IJ . RIGging an antiviral defense—it’s in the CARDs. Nat Immunol. (2004) 5:699–701. doi: 10.1038/ni0704-699 15224097

[26] Onomoto K Onoguchi K Yoneyama M . Regulation of RIG-I-like receptor-mediated signaling: interaction between host and viral factors. Cell Mol Immunol. (2021) 18:539. doi: 10.1038/s41423-020-00602-7 33462384 PMC7812568

[27] Dvorkin S Cambier S Volkman HE Stetson DB . New frontiers in the cGAS-STING intracellular DNA-sensing pathway. Immunity. (2024) 57:718–30. doi: 10.1016/j.immuni.2024.02.019 38599167 PMC11013568

[28] Lu Y Zhao M Chen L Wang Y Liu T Liu H . cGAS: action in the nucleus. Front Immunol. (2024) 15:1380517. doi: 10.3389/fimmu.2024.1380517 38515746 PMC10954897

[29] Nelson TS Ma Z . STING agonists as antiviral agents. FEBS Lett. (2025) 600(8):1151–84. doi: 10.1002/1873-3468.70251 41406030 PMC12831998

[30] Liu S Cai X Wu J Cong Q Chen X Li T . Phosphorylation of innate immune adaptor proteins MAVS, STING, and TRIF induces IRF3 activation. Sci (1979). (2015) 347. doi: 10.1126/science.aaa2630 25636800

[31] Amurri L Horvat B Iampietro M . Interplay between RNA viruses and cGAS/STING axis in innate immunity. Front Cell Infect Microbiol. (2023) 13:1172739. doi: 10.3389/fcimb.2023.1172739 37077526 PMC10106766

[32] Chen R Zou J Chen J Zhong X Kang R Tang D . Pattern recognition receptors: function, regulation and therapeutic potential. Signal Transduct Target Ther. (2025) 10:216. doi: 10.1038/s41392-025-02264-1 40640149 PMC12246121

[33] Madiraju C Novack JP Reed JC Matsuzawa S . K63 ubiquitination in immune signaling. Trends Immunol. (2022) 43:148–62. doi: 10.1016/j.it.2021.12.005 35033428 PMC8755460

[34] Zhang J Hu MM Wang YY Shu HB . TRIM32 protein modulates type I interferon induction and cellular antiviral response by targeting MITA/STING protein for K63-linked ubiquitination. J Biol Chem. (2012) 287:28646–55. doi: 10.1074/jbc.M112.362608 22745133 PMC3436586

[35] Zheng Y Gao C . E3 ubiquitin ligases, the powerful modulator of innate antiviral immunity. Cell Immunol. (2019) 340:103915. doi: 10.1016/j.cellimm.2019.04.003 31054776

[36] Liu S Chen ZJ . Expanding role of ubiquitination in NF-κB signaling. Cell Res. (2010) 21:6–21. doi: 10.1038/cr.2010.170 21135871 PMC3193409

[37] Ohtake F Saeki Y Ishido S Kanno J Tanaka K . The K48-K63 branched ubiquitin chain regulates NF-κB signaling. Mol Cell. (2016) 64:251–66. doi: 10.1016/J.MOLCEL.2016.09.014 27746020

[38] Oshiumi H . Recent advances and contradictions in the study of the individual roles of ubiquitin ligases that regulate RIG-I-like receptor-mediated antiviral innate immune responses. Front Immunol. (2020) 11:549369. doi: 10.3389/fimmu.2020.01296 PMC7326816 32670286

[39] Gu Z Chen X Yang W Qi Y Yu H Wang X . The SUMOylation of TAB2 mediated by TRIM60 inhibits MAPK/NF-κB activation and the innate immune response. Cell Mol Immunol. (2020) 18:1981–94. doi: 10.1038/s41423-020-00564-w 33184450 PMC8322076

[40] Sarkar L Liu GQ Acharya D Zhu J Sayyad Z Gack MU . MDA5 ISGylation is crucial for immune signaling to control viral replication and pathogenesis. Proc Natl Acad Sci USA. (2025) 122:e2420190122. doi: 10.1073/PNAS.2420190122 40184173 PMC12002354

[41] Li Q Lin L Tong Y Liu Y Mou J Wang X . TRIM29 negatively controls antiviral immune response through targeting STING for degradation. Cell Discov. (2018) 4:13. doi: 10.1038/S41421-018-0010-9 29581886 PMC5859251

[42] Xing J Zhang A Minze LJ Li XC Zhang Z . TRIM29 negatively regulates the type I IFN production in response to RNA virus. J Immunol. (2018) 201:183–92. doi: 10.4049/JIMMUNOL.1701569 29769269 PMC6092021

[43] Wang J Wang L Lu W Farhataziz N Gonzalez A Xing J . TRIM29 controls enteric RNA virus-induced intestinal inflammation by targeting NLRP6 and NLRP9b signaling pathways. Mucosal Immunol. (2025) 18:135–50. doi: 10.1016/j.mucimm.2024.10.004 39396665 PMC12210021

[44] Xing J Zhang A Zhang H Wang J Li XC Zeng MS . TRIM29 promotes DNA virus infections by inhibiting innate immune response. Nat Commun. (2017) 8:945. doi: 10.1038/s41467-017-00101-w 29038422 PMC5643338

[45] Wang J Lu W Zhang J Du Y Fang M Zhang A . Loss of TRIM29 mitigates viral myocarditis by attenuating PERK-driven ER stress response in male mice. Nat Commun. (2024) 15:3481. doi: 10.1038/s41467-024-44745-x 38664417 PMC11045800

[46] Xing J Weng L Yuan B Wang Z Jia L Jin R . Identification of a role for TRIM29 in the control of innate immunity in the respiratory tract. Nat Immunol. (2016) 17:1373–80. doi: 10.1038/ni.3580 27695001 PMC5558830

[47] Zong Z Zhang Z Wu L Zhang L Zhou F . The functional deubiquitinating enzymes in control of innate antiviral immunity. Adv Sci. (2020) 8:2002484. doi: 10.1002/advs.202002484 33511009 PMC7816709

[48] Zhang Q Jia Q Gao W Zhang W . The role of deubiquitinases in virus replication and host innate immune response. Front Microbiol. (2022) 13:839624. doi: 10.3389/fmicb.2022.839624 35283827 PMC8908266

[49] Bailey-Elkin BA Knaap RCM Kikkert M Mark BL . Structure and function of viral deubiquitinating enzymes. J Mol Biol. (2017) 429:3441–70. doi: 10.1016/j.jmb.2017.06.010 28625850 PMC7094624

[50] Cao D Duan L Huang B Xiong Y Zhang G Huang H . The SARS-CoV-2 papain-like protease suppresses type I interferon responses by deubiquitinating STING. Sci Signal. (2023) 16. doi: 10.1126/scisignal.add0082 37130168

[51] Akutsu M Ye Y Virdee S Chin JW Komander D . Molecular basis for ubiquitin and ISG15 cross-reactivity in viral ovarian tumor domains. Proc Natl Acad Sci USA. (2011) 108:2228–33. doi: 10.1073/pnas.1015287108 21266548 PMC3038727

[52] Guven-Maiorov E Tsai CJ Nussinov R . Pathogen mimicry of host protein-protein interfaces modulates immunity. Semin Cell Dev Biol. (2016) 58:136–45. doi: 10.1016/j.semcdb.2016.06.004 27287306

[53] Goswami S Samanta D Duraivelan K . Molecular mimicry of host short linear motif-mediated interactions utilised by viruses for entry. Mol Biol Rep. (2023) 50:4665. doi: 10.1007/s11033-023-08389-2 37016039 PMC10072811

[54] Mansur DS Maluquer de Motes C Unterholzner L Sumner RP Ferguson BJ Ren H . Poxvirus targeting of E3 ligase β-TrCP by molecular mimicry: a mechanism to inhibit NF-κB activation and promote immune evasion and virulence. PloS Pathog. (2013) 9:e1003183. doi: 10.1371/journal.ppat.1003183 23468625 PMC3585151

[55] Querido E Blanchette P Yan Q Kamura T Morrison M Boivin D . Degradation of p53 by adenovirus E4orf6 and E1B55K proteins occurs via a novel mechanism involving a Cullin-containing complex. Genes Dev. (2001) 15:3104–17. doi: 10.1101/gad.926401 11731475 PMC312842

[56] Luftig M Yasui T Soni V Kang MS Jacobson N Cahir-McFarland E . Epstein–Barr virus latent infection membrane protein 1 TRAF-binding site induces NIK/IKKα-dependent noncanonical NF-κB activation. Proc Natl Acad Sci. (2004) 101:141–6. doi: 10.1073/pnas.2237183100 14691250 PMC314152

[57] Munir A Khan S Saleem A Nusrat H Khan SA Sayyed H . The role of Epstein–Barr virus molecular mimicry in various autoimmune diseases. Scand J Immunol. (2025) 101:e70016. doi: 10.1111/sji.70016 40155782

[58] Maguire C Wang C Ramasamy A Fonken C Morse B Lopez N . Molecular mimicry as a mechanism of viral immune evasion and autoimmunity. Nat Commun. (2024) 15:9403. doi: 10.1038/s41467-024-53658-8 39477943 PMC11526117

[59] Ramazi S Zahiri J . Post-translational modifications in proteins: resources, tools and prediction methods. Database. (2021) 2021. doi: 10.1093/DATABASE/BAAB012 33826699 PMC8040245

[60] Medvedev KE Schaeffer RD Grishin NV . Leveraging AI to explore structural contexts of post-translational modifications in drug binding. J Cheminf. (2025) 17:67. doi: 10.1186/S13321-025-01019-Y 40320551 PMC12051291

[61] Fu H Yang Y Wang X Wang H Xu Y . DeepUbi: a deep learning framework for prediction of ubiquitination sites in proteins. BMC Bioinf. (2019) 20:86. doi: 10.1186/s12859-019-2677-9 30777029 PMC6379983

[62] Yadav S Gupta M Bist AS . Prediction of ubiquitination sites using UbiNets. Adv Fuzzy Syst. (2018) 2018:5125103. doi: 10.1155/2018/5125103

[63] He F Wang R Li J Bao L Xu D Zhao X . Large-scale prediction of protein ubiquitination sites using a multimodal deep architecture. BMC Syst Biol. (2018) 12:109. doi: 10.1186/s12918-018-0628-0 30463553 PMC6249717

[64] Wang JR Huang WL Tsai MJ Hsu KT Huang HL Ho SY . ESA-UbiSite: accurate prediction of human ubiquitination sites by identifying a set of effective negatives. Bioinformatics. (2017) 33:661–8. doi: 10.1093/bioinformatics/btw701 28062441

[65] Abramson J Adler J Dunger J Evans R Green T Pritzel A . Accurate structure prediction of biomolecular interactions with AlphaFold 3. Nature. (2024) 630:493–500. doi: 10.1038/s41586-024-07487-w 38718835 PMC11168924

[66] Peng C Ni W Liu Q Hu G Zheng W . A comprehensive benchmarking of the AlphaFold3 for predicting biomacromolecules and their interactions. Brief Bioinform. (2025) 26. doi: 10.1093/bib/bbaf616 41313605 PMC12661943

[67] Fábián B Stuke JFM Heinz M Hummer G . AlphaFold modeling of polyubiquitin complexes and covalently linked proteins. Cell Rep Phys Sci. (2025) 6:102796. doi: 10.1016/J.XCRP.2025.102796 38826717

[68] Humphreys IR Zhang J Baek M Wang Y Krishnakumar A Pei J . Protein interactions in human pathogens revealed through deep learning. Nat Microbiol. (2024) 9:2642–52. doi: 10.1038/s41564-024-01791-x 39294458 PMC11445079

[69] Bonardi A . Computational approaches for designing viral protease inhibitors. Enzymes (Essen). (2025) 58:59–91. doi: 10.1016/bs.enz.2025.06.005 41238303

[70] Kawai K Nagata N Takahashi Y . De novo design of drug-like molecules by a fragment-based molecular evolutionary approach. J Chem Inf Model. (2014) 54:49–56. doi: 10.1021/ci400418c 24372539

[71] Ferruz N Stein A . Computational methods for protein design. Protein Eng Des Sel. (2024) 37. doi: 10.1093/PROTEIN/GZAE011 38984793

[72] Albanese KI Barbe S Tagami S Woolfson DN Schiex T . Computational protein design. Nat Rev Methods Primers. (2025) 5:13. doi: 10.1038/s43586-025-00383-1 37880705

[73] Li Y Pei J Lai L . Structure-based de novo drug design using 3D deep generative models. Chem Sci. (2021) 12:13664–75. doi: 10.1039/D1SC04444C 34760151 PMC8549794

[74] Gupta R Srivastava D Sahu M Tiwari S Ambasta RK Kumar P . Artificial intelligence to deep learning: machine intelligence approach for drug discovery. Mol Divers. (2021) 25:1315. doi: 10.1007/s11030-021-10217-3 33844136 PMC8040371

[75] Dara S Dhamercherla S Jadav SS Babu CM Ahsan MJ . Machine learning in drug discovery: a review. Artif Intell Rev. (2022) 55:1947–99. doi: 10.1007/s10462-021-10058-4 34393317 PMC8356896

[76] Popova M Isayev O Tropsha A . Deep reinforcement learning for de novo drug design. Sci Adv. (2018) 4. doi: 10.1126/sciadv.aap7885 30050984 PMC6059760

[77] Chen Y Xue W . Machine learning for de novo molecular generation: a comprehensive review. ACS Chem Neurosci. (2026) 17:666–80. doi: 10.1021/acschemneuro.5c00861 41665230

[78] Wang M Wang Z Sun H Wang J Shen C Weng G . Deep learning approaches for de novo drug design: an overview. Curr Opin Struct Biol. (2022) 72:135–44. doi: 10.1016/j.sbi.2021.10.001 34823138

[79] Chakraborty A Krishnan V Thamotharan S . Generative adversarial network (GAN) model-based design of potent SARS-CoV-2 Mpro inhibitors using the electron density of ligands and 3D binding pockets: insights from molecular docking, dynamics simulation, and MM-GBSA analysis. Mol Divers. (2025) 29:3059–75. doi: 10.1007/s11030-024-11047-9 39613993

[80] Arshia AH Shadravan S Solhjoo A Sakhteman A Sami A . De novo design of novel protease inhibitor candidates in the treatment of SARS-CoV-2 using deep learning, docking, and molecular dynamic simulations. Comput Biol Med. (2021) 139:104967. doi: 10.1016/j.compbiomed.2021.104967 34739968 PMC8545757

[81] Müller AT Hiss JA Schneider G . Recurrent neural network model for constructive peptide design. J Chem Inf Model. (2018) 58:472–9. doi: 10.1021/acs.jcim.7b00414 29355319

[82] Yunxiang Y Zhou Z Hai G Xinlu R Yuting Z Jianna M . Ai-driven de novo design of customizable membrane permeable cyclic peptides. J Comput-Aided Mol Des. (2025) 39:63. doi: 10.1007/s10822-025-00639-8 40782270

[83] García EG Varas Pardo P González-Naranjo P Ulzurrun E Marcos-Ayuso G Pérez C . Correction to “AI-driven de novo design and development of nontoxic DYRK1A inhibitors. J Med Chem. (2025) 68:26592. doi: 10.1021/acs.jmedchem.5c02889 41358654 PMC12751003

[84] Tang Y Moretti R Meiler J . Recent advances in automated structure-based de novo drug design. J Chem Inf Model. (2024) 64:1794–805. doi: 10.1021/ACS.JCIM.4C00247 38485516 PMC10966644

[85] Ma Z Zhou M Chen H Shen Q Zhou J . Deubiquitinase-targeting chimeras (DUBTACs) as a potential paradigm-shifting drug discovery approach. J Med Chem. (2025) 68:6897. doi: 10.1021/acs.jmedchem.4c02975 40135978 PMC12507175

[86] Liu Z Hu M Yang Y Du C Zhou H Liu C . An overview of PROTACs: a promising drug discovery paradigm. Mol BioMed. (2022) 3:46. doi: 10.1186/S43556-022-00112-0 36536188 PMC9763089

[87] Islam S Hussain A Alam A Ansari MO Saeed H Khan S . Editorial: The ubiquitin-proteasome system and cellular signaling: mechanisms and regulatory roles in cancer and infectious diseases. Front Cell Dev Biol. (2026) 14:1812881. doi: 10.3389/FCELL.2026.1812881 41822349 PMC12975994

[88] Zhong G Chang X Xie W Zhou X . Targeted protein degradation: advances in drug discovery and clinical practice. Signal Transduction Targeted Ther. (2024) 9:308. doi: 10.1038/s41392-024-02004-x 39500878 PMC11539257

[89] Bhole RP Labhade S Gurav SS . Conquering PROTAC molecular design and drugability. Bioanalysis. (2025) 17:455–70. doi: 10.1080/17576180.2025.2481021 40114295 PMC12026086

[90] Tan S Chen Z Lu R Liu H Yao X . Rational proteolysis targeting chimera design driven by molecular modeling and machine learning. Wiley Interdiscip Rev Comput Mol Sci. (2025) 15. doi: 10.1002/wcms.70013 41531421

[91] Li F Hu Q Zhang X Sun R Liu Z Wu S . DeepPROTACs is a deep learning-based targeted degradation predictor for PROTACs. Nat Commun. (2022) 13:7133. doi: 10.1038/s41467-022-34807-3 36414666 PMC9681730

[92] Liu J Roy MJ Isbel L Li F . Accurate PROTAC-targeted degradation prediction with DegradeMaster. Bioinformatics. (2025) 41:i342–51. doi: 10.1093/BIOINFORMATICS/BTAF191 40662822 PMC12261415

[93] Xie L Xie L . Elucidation of genome-wide understudied proteins targeted by PROTAC-induced degradation using interpretable machine learning. PloS Comput Biol. (2023) 19:e1010974. doi: 10.1371/journal.pcbi.1010974 37590332 PMC10464998

[94] Schönfeld J Liebisch N Brunst S Weizel L Knapp S Kannt A . Click chemistry enables rapid development of potent sEH PROTACs using a direct-to-biology approach. Chem Commun. (2025) 61:18108–11. doi: 10.1039/d5cc03325j 41120092

[95] McCorkindale W Filep M London N Lee AA King-Smith E . Deconvoluting low yield from weak potency in direct-to-biology workflows with machine learning. RSC Med Chem. (2024) 15:1015–21. doi: 10.1039/d3md00719g 38516605 PMC10953487

[96] Bhole RP Kute P Gurav SS . PROTACs in the treatment of viral diseases. Future Med Chem. (2025) 17:267. doi: 10.1080/17568919.2025.2453418 39814466 PMC11792865

[97] Sasso JM Tenchov R Wang DS Johnson LS Wang X Zhou QA . Molecular glues: the adhesive connecting targeted protein degradation to the clinic. Biochemistry. (2022) 62:601. doi: 10.1021/acs.biochem.2c00245 35856839 PMC9910052

[98] Smith MC Gestwicki JE . Features of protein-protein interactions that translate into potent inhibitors: topology, surface area and affinity. Expert Rev Mol Med. (2012) 14:e16. doi: 10.1017/erm.2012.10 22831787 PMC3591511

[99] Rodrigues CHM Pires DEV Blundell TL Ascher DB . Structural landscapes of PPI interfaces. Brief Bioinform. (2022) 23:bbac165. doi: 10.1093/bib/bbac165 35656714 PMC9294409

[100] Zhang C Liu Y Li G Yang Z Han C Sun X . Targeting the undruggables—the power of protein degraders. Sci Bull (Beijing). (2024) 69:1776–97. doi: 10.1016/j.scib.2024.03.056 38614856

[101] Liu Y Bai J Li D Cang Y . Routes to molecular glue degrader discovery. Trends Biochem Sci. (2025) 50:134–42. doi: 10.1016/j.tibs.2024.12.006 39753433

[102] Tan X Huang Z Pei H Jia Z Zheng J . Molecular glue-mediated targeted protein degradation: a novel strategy in small-molecule drug development. Iscience. (2024) 27:110712. doi: 10.1016/j.isci.2024.110712 39297173 PMC11409024

[103] Kozicka Z Suchyta DJ Focht V Kempf G Petzold G Jentzsch M . Design principles for cyclin K molecular glue degraders. Nat Chem Biol. (2023) 20:93–102. doi: 10.1038/s41589-023-01409-z 37679459 PMC10746543

[104] Wang J Mao J Li C Xiang H Wang X Wang S . Interface-aware molecular generative framework for protein–protein interaction modulators. J Cheminform. (2024) 16:142. doi: 10.1186/s13321-024-00930-0 39707457 PMC11662471

[105] Chakravarty A Yang PL . Targeted protein degradation as an antiviral approach. Antiviral Res. (2022) 210:105480. doi: 10.1016/J.ANTIVIRAL.2022.105480 36567024 PMC10178900

[106] Gosavi PM Ngan KC Yeo MJR Su C Li J Lue NZ . Profiling the landscape of drug resistance mutations in neosubstrates to molecular glue degraders. ACS Cent Sci. (2022) 8:417–29. doi: 10.1021/ACSCENTSCI.1C01603 35505873 PMC9052798

[107] Ma L Wang N Zhu J Wu L He S Zhang B . Molecular glue degraders: rational design, specificity engineering, and advanced delivery. Acta Pharm Sin B. (2026). doi: 10.1016/J.APSB.2026.02.020 38826717

[108] Qamar T Bawany NZ . Understanding the black-box: towards interpretable and reliable deep learning models. PeerJ Comput Sci. (2023) 9:e1629. doi: 10.7717/peerj-cs.1629 38077598 PMC10702969

[109] Ali S Abuhmed T El-Sappagh S Muhammad K Alonso-Moral JM Confalonieri R . Explainable artificial intelligence (XAI): what we know and what is left to attain trustworthy artificial intelligence. Inf Fusion. (2023) 99:101805. doi: 10.1016/J.INFFUS.2023.101805 38826717

[110] Mohapatra RK Jolly L Dakua SP . Advancing explainable AI in healthcare: necessity, progress, and future directions. Comput Biol Chem. (2025) 119:108599. doi: 10.1016/J.COMPBIOLCHEM.2025.108599 40743677

[111] van Royen FS Weerts HJP de Hond AAH Geersing GJ Rutten FH Moons KGM . In humble defense of unexplainable black box prediction models in healthcare. J Clin Epidemiol. (2026) 189. doi: 10.1016/j.jclinepi.2025.112013 41077324

[112] Silva JCF Schuster L Sexson N Erdem M Hulke R Kirst M . InteracTor: feature engineering and explainable AI for profiling protein structure-interaction-function relationships. PloS Comput Biol. (2025) 21:e1013038. doi: 10.1371/JOURNAL.PCBI.1013038 41082567 PMC12614802

[113] Wenzel M Grüner E Strodthoff N . Insights into the inner workings of transformer models for protein function prediction. Bioinformatics. (2024) 40:btae031. doi: 10.1093/BIOINFORMATICS/BTAE031 38244570 PMC10950482

[114] Xiong A Luo Z Xia Y Zou Q Wei L Zhang Z . An interpretable geometric graph neural network for enhancing the generalizability of drug–target interaction prediction. BMC Biol. (2025) 23:350. doi: 10.1186/S12915-025-02456-9 41299450 PMC12659342

[115] Chen Z Gu C Tan S Wang X Li Y He M . Interpretable PROTAC degradation prediction with structure‐informed deep ternary attention framework. Adv Sci. (2025) 12:e08138. doi: 10.1002/ADVS.202508138 41026144 PMC12713099

[116] Sadeghi Z Alizadehsani R Cifci MA Kausar S Rehman R Mahanta P . A review of explainable artificial intelligence in healthcare. Comput Electr Eng. (2024) 118:109370. doi: 10.1016/J.COMPELECENG.2024.109370 38826717

[117] Zhou C Yang S Wang J Pan W Yao H Li G . Recent advances in PROTAC-based antiviral and antibacterial therapeutics. Bioorg Chem. (2025) 160:108437. doi: 10.1016/J.BIOORG.2025.108437 40215946

[118] Békés M Langley DR Crews CM . PROTAC targeted protein degraders: the past is prologue. Nat Rev Drug Discov. (2022) 21:181–200. doi: 10.1038/S41573-021-00371-6 35042991 PMC8765495

[119] Tirosyan I Gabrielyan Y Petrosyan V Vignuzzi M Zakaryan H . Can artificial intelligence transform antiviral drug discovery? Drug Discov Today. (2026) 31:104648. doi: 10.1016/J.DRUDIS.2026.104648 41875943

[120] Anh-Hoang D Tran V Nguyen LM . Survey and analysis of hallucinations in large language models: attribution to prompting strategies or model behavior. Front Artif Intell. (2025) 8:1622292. doi: 10.3389/FRAI.2025.1622292 41098969 PMC12518350

[121] Lecu A Groza A Hawizy L . Reducing hallucinations in medical AI: a knowledge graph-augmented retrieval system for evidence-based age-related macular degeneration information. IEEE Access. (2025) 13:210624–39. doi: 10.1109/ACCESS.2025.3643370 25079929

[122] Chen F Li Y Chen Y Bian Z Duo L Zhou Q . Strategies for the analysis and elimination of hallucinations in artificial intelligence generated medical knowledge. J Evid Based Med. (2025) 18:e70075. doi: 10.1111/JEBM.70075 40983876

[123] Latham AP Zhang W Tempkin JOB Otsuka S Ellenberg J Sali A . Integrative spatiotemporal modeling of biomolecular processes: application to the assembly of the nuclear pore complex. Proc Natl Acad Sci USA. (2025) 122:e2415674122. doi: 10.1073/PNAS.2415674122 40085653 PMC11929490

[124] Cui X Ge L Chen X Lv Z Wang S Zhou X . Beyond static structures: protein dynamic conformations modeling in the post-AlphaFold era. Brief Bioinform. (2025) 26. doi: 10.1093/BIB/BBAF340 40663654 PMC12262120

[125] Li Y Zhan RH Rao J Liu M Sang P Zeng X . Structure-informed machine learning for drug discovery: a task-centric perspective. Brief Bioinform. (2026) 27. doi: 10.1093/BIB/BBAG081 41729820 PMC12927881

[126] Mao Y . Dynamics-based drug discovery by time-resolved cryo-EM. Curr Opin Struct Biol. (2025) 91:103001. doi: 10.1016/J.SBI.2025.103001 39985947

[127] Zhang S Zou S Yin D Zhao L Finley D Wu Z . USP14-regulated allostery of the human proteasome by time-resolved cryo-EM. Nature. (2022) 605:567–74. doi: 10.1038/s41586-022-04671-8 35477760 PMC9117149

[128] Huang Z Cui X Xia Y Zhao K Zhang G . Pathfinder: protein folding pathway prediction based on conformational sampling. PloS Comput Biol. (2023) 19:e1011438. doi: 10.1371/JOURNAL.PCBI.1011438 37695768 PMC10513300

[129] Zhang Y Skolnick J . SPICKER: a clustering approach to identify near-native protein folds. J Comput Chem. (2004) 25:865–71. doi: 10.1002/JCC.20011 15011258

[130] Liu Y Li D Zhang X Xia S Qu Y Ling X . A protein sequence-based deep transfer learning framework for identifying human proteome-wide deubiquitinase-substrate interactions. Nat Commun. (2024) 15:4519. doi: 10.1038/s41467-024-48446-3 38806474 PMC11133436

[131] Ge F Zhang L Hou YF Chen Y Ullah A Dral PO . Four-dimensional-spacetime atomistic artificial intelligence models. J Phys Chem Lett. (2023) 14:7732–43. doi: 10.1021/ACS.JPCLETT.3C01592 37606602

[132] Wang T He X Li M Li Y Bi R Wang Y . Ab initio characterization of protein molecular dynamics with AI2BMD. Nature. (2024) 635:1019–27. doi: 10.1038/s41586-024-08127-z 39506110 PMC11602711

[133] Maelfait J Beyaert R . Emerging role of ubiquitination in antiviral RIG-I signaling. Microbiol Mol Biol Rev. (2012) 76:33–45. doi: 10.1128/MMBR.05012-11 22390971 PMC3294425

[134] Zhou Y He C Wang L Ge B . Post-translational regulation of antiviral innate signaling. Eur J Immunol. (2017) 47:1414–26. doi: 10.1002/EJI.201746959 28744851 PMC7163624

[135] Tang Y Joo D Liu G Tu H You J Jin J . Linear ubiquitination of cFLIP induced by LUBAC contributes to TNFα-induced apoptosis. J Biol Chem. (2018) 293:20062–72. doi: 10.1074/JBC.RA118.005449 30361438 PMC6311529

[136] Fonseca D Pisanelli G Seoane R Miorin L García-Sastre A . TRIM65 regulates innate immune signaling by enhancing K6-linked ubiquitination of IRF3 and its chromatin recruitment. Cell Rep. (2024) 43:114960. doi: 10.1016/J.CELREP.2024.114960 39580801

[137] van Huizen M Kikkert M . The role of atypical ubiquitin chains in the regulation of the antiviral innate immune response. Front Cell Dev Biol. (2020) 7:502312. doi: 10.3389/FCELL.2019.00392 32039206 PMC6987411

[138] Xue B Li H Guo M Wang J Xu Y Zou X . TRIM21 promotes innate immune response to RNA viral infection through Lys27-linked polyubiquitination of MAVS. J Virol. (2018) 92:e00321-18. doi: 10.1128/JVI.00321-18 29743353 PMC6026736

[139] Shao T Pei Z Wang Y Zhao Y Fan H Pan J . The roles of post-translational modifications in the pathogenesis of RNA viruses: allies or adversaries? Front Microbiol. (2026) 17:1768721. doi: 10.3389/FMICB.2026.1768721 41767575 PMC12936031

[140] Rhamadianti AF Abe T Tanaka T Ono C Katayama H Makino Y . SARS-CoV-2 papain-like protease inhibits ISGylation of the viral nucleocapsid protein to evade host anti-viral immunity. J Virol. (2024) 98. doi: 10.1128/JVI.00855-24 39120134 PMC11406913

[141] Fan Y Li X Zhang L Zong Z Wang F Huang J . SUMOylation in viral replication and antiviral defense. Adv Sci. (2022) 9:2104126. doi: 10.1002/ADVS.202104126 35060688 PMC8895153

[142] Wang G Zhao Y Zhou Y Jiang L Liang L Kong F . PIAS1-mediated SUMOylation of influenza A virus PB2 restricts viral replication and virulence. PloS Pathog. (2022) 18. doi: 10.1371/JOURNAL.PPAT.1010446 35377920 PMC9009768

[143] Zhu J Chu F Zhang M Sun W Zhou F . Association between neddylation and immune response. Front Cell Dev Biol. (2022) 10:890121. doi: 10.3389/FCELL.2022.890121 35602593 PMC9117624

[144] Radivojac P Vacic V Haynes C Cocklin RR Mohan A Heyen JW . Identification, analysis, and prediction of protein ubiquitination sites. Proteins: Structure Funct Bioinf. (2010) 78:365–80. doi: 10.1002/PROT.22555 19722269 PMC3006176

[145] Wang X Li Y He M Kong X Jiang P Liu X . UbiBrowser 2.0: a comprehensive resource for proteome-wide known and predicted ubiquitin ligase/deubiquitinase–substrate interactions in eukaryotic species. Nucleic Acids Res. (2022) 50:D719–28. doi: 10.1093/NAR/GKAB962 34669962 PMC8728189

[146] Van Zundert GCP Rodrigues JPGLM Trellet M Schmitz C Kastritis PL Karaca E . The HADDOCK2.2 web server: user-friendly integrative modeling of biomolecular complexes. J Mol Biol. (2016) 428:720–5. doi: 10.1016/J.JMB.2015.09.014 26410586

[147] Yin R Feng BY Varshney A Pierce BG . Benchmarking AlphaFold for protein complex modeling reveals accuracy determinants. Protein Sci. (2022) 31:e4379. doi: 10.1002/PRO.4379 35900023 PMC9278006

[148] Zhang J Humphreys IR Pei J Kim J Choi C Yuan R . Predicting protein-protein interactions in the human proteome. Sci (1979). (2025) 390. doi: 10.1126/SCIENCE.ADT1630 40997207 PMC13281779

[149] Zhavoronkov A Ivanenkov YA Aliper A Veselov MS Aladinskiy VA Aladinskaya AV . Deep learning enables rapid identification of potent DDR1 kinase inhibitors. Nat Biotechnol. (2019) 37:1038–40. doi: 10.1038/S41587-019-0224-X 31477924

[150] Pakhrin SC Beck MR Subedi P Lama R Shrestha S . Multimodal deep learning for predicting protein ubiquitination sites. Bioinf Adv. (2024) 5. doi: 10.1093/BIOADV/VBAF200 40917649 PMC12408473

[151] Sun J Sun D Yang Q Wang D Peng J Guo H . A novel, covalent broad-spectrum inhibitor targeting human coronavirus Mpro. Nat Commun. (2025) 16:4546. doi: 10.1038/s41467-025-59870-4 40374668 PMC12081877

[152] Ivanenkov YA Polykovskiy D Bezrukov D Zagribelnyy B Aladinskiy V Kamya P . Chemistry42: An AI-driven platform for molecular design and optimization. J Chem Inf Model. (2023) 63:695–701. doi: 10.1021/ACS.JCIM.2C01191 36728505 PMC9930109

[153] Li H Wang S Ma W Cheng B Yi Y Ma X . Discovery of pentacyclic triterpenoid PROTACs as a class of effective hemagglutinin protein degraders. J Med Chem. (2022) 65:7154–69. doi: 10.1021/ACS.JMEDCHEM.1C02013 35579113

[154] Hemant Kumar S Venkatachalapathy M Sistla R Poongavanam V . Advances in molecular glues: exploring chemical space and design principles for targeted protein degradation. Drug Discov Today. (2024) 29:104205. doi: 10.1016/J.DRUDIS.2024.104205 39393773

[155] Ben Geoffrey AS Agrawal D Kulkarni NM Gunasekaran M . Molecular glue-design-evaluator (MOLDE): An advanced method for in-silico molecular glue design. ACS Omega. (2025) 10:6650–62. doi: 10.1021/ACSOMEGA.4C08049 40028145 PMC11865985

[156] Wan F Kontogiorgos-Heintz D de la Fuente Nunez C . Deep generative models for peptide design. Digital Discov. (2022) 1:195–208. doi: 10.1039/D1DD00024A 35769205 PMC9189861

[157] Surana S Arora P Singh D Sahasrabuddhe D Valadi J . PandoraGAN: Generating antiviral peptides using generative adversarial network. SN Comput Sci. (2023) 4:607. doi: 10.1007/S42979-023-02203-3 30311153

[158] Sanders JM Monogue ML Jodlowski TZ Cutrell JB . Pharmacologic treatments for coronavirus disease 2019 (COVID-19): A review. JAMA. (2020) 323:1824–36. doi: 10.1001/JAMA.2020.6019 32282022

[159] Zhou Y Wang F Tang J Nussinov R Cheng F . Artificial intelligence in COVID-19 drug repurposing. Lancet Digit Health. (2020) 2:e667–76. doi: 10.1016/S2589-7500(20)30192-8 32984792 PMC7500917

